# In_2_O_3_: An Oxide Semiconductor for Thin-Film Transistors, a Short Review

**DOI:** 10.3390/molecules30244762

**Published:** 2025-12-12

**Authors:** Christophe Avis, Jin Jang

**Affiliations:** Department of Information Display, Advanced Display Research Center, Kyung Hee University, Seoul 02447, Republic of Korea

**Keywords:** In_2_O_3_, thin-film transistors, doping, neuromorphic applications, 3D integration, ITO, IGO, ALD, spray-pyrolysis

## Abstract

With the discovery of amorphous oxide semiconductors, a new era of electronics opened. Indium gallium zinc oxide (IGZO) overcame the problems of amorphous and poly-silicon by reaching mobilities of ~10 cm^2^/Vs and demonstrating thin-film transistors (TFTs) are easy to manufacture on transparent and flexible substrates. However, mobilities over 30 cm^2^/Vs have been difficult to reach and other materials have been introduced. Recently, polycrystalline In_2_O_3_ has demonstrated breakthroughs in the field. In_2_O_3_ TFTs have attracted attention because of their high mobility of over 100 cm^2^/Vs, which has been achieved multiple times, and because of their use in scaled devices with channel lengths down to 10 nm for high integration in back-end-of-the-line (BEOL) applications and others. The present review focuses first on the material properties with the understanding of the bandgap value, the importance of the position of the charge neutrality level (CNL), the doping effect of various atoms (Zr, Ge, Mo, Ti, Sn, or H) on the carrier concentration, the optical properties, the effective mass, and the mobility. We introduce the effects of the non-parabolicity of the conduction band and how to assess them. We also introduce ways to evaluate the CNL position (usually at ~E_C_ + 0.4 eV). Then, we describe TFTs’ general properties and parameters, like the field effect mobility, the subthreshold swing, the measurements necessary to assess the TFT stability through positive and negative bias temperature stress, and the negative bias illumination stress (NBIS), to finally introduce In_2_O_3_ TFTs. Then, we will introduce vacuum and non-vacuum processes like spin-coating and liquid metal printing. We will introduce the various dopants and their applications, from mobility and crystal size improvements with H to NBIS improvements with lanthanides. We will also discuss the importance of device engineering, introducing how to choose the passivation layer, the source and drain, the gate insulator, the substrate, but also the possibility of advanced engineering by introducing the use of dual gate and 2 DEG devices on the mobility improvement. Finally, we will introduce the recent breakthroughs where In_2_O_3_ TFTs are integrated in neuromorphic applications and 3D integration.

## 1. Introduction

Amorphous oxide semiconductors (AOS) have attracted attention since 2004 with the discovery of a-IGZO as a viable semiconductor for TFT applications with mobilities of ~10 cm^2^/Vs [1]. Oxide semiconductors are made of oxygen and post-transition metal cations ((n − 1)d^10^ ns, n ≥ 4). The oxygen p orbitals mostly form the top of the valence band, and the ns form the bottom of the conduction band. The overlap of the ns orbitals easily makes the electron pathway. The spherical s orbitals are less prone to a direction preference than the sp^3^ orbitals of Si, therefore the amorphous oxide semiconductors are less prone to electron mobility degradation than the amorphous Si is relative to the crystalline Si [1]. The curvature of the conduction band minimum (CBM) demonstrates the low effective mass of the electrons, leading to high mobilities, whereas the flat curvature of the valence band maximum (VBM) translates into heavy hole and low mobility. This is one of the reasons why high mobility p-type oxide semiconductors are difficult to achieve [2,3]. The oxide semiconductors usually have a bandgap of ≥3 eV and are therefore transparent in the visible region.

Oxide semiconductors are intrinsically n-type semiconductors, due, in part, to the presence of ionized oxygen vacancies providing extra electrons [1]. Electron conduction follows a percolation mechanism through potential barriers, so the higher the carrier concentration leads to a higher mobility of the charge carriers [4].

Other than IGZO, other AOS have been developed. Two cations are usually used to maintain an amorphous phase, like, for example, IZO, IGO, ZTO, or IZTO [1,5]. Polycrystalline oxide materials have been investigated too. Indeed, they usually provide higher TFT performances. Among them, ZnO, SnO_2_, and In_2_O_3_ have been under extensive research. While In_2_O_3_ and SnO_2_ may show similar device performances, the process difficulties related to SnO_2_ may make In_2_O_3_ a more attractive solution for high-performance device manufacturing. Especially with the emergence of pseudo-CMOS applications like low-temperature polysilicon oxide (LTPO), [6] high-mobility oxide TFTs are required, and TFTs based on In_2_O_3_ or doped In_2_O_3_ could represent a viable solution. Yet the reader needs to keep in mind that TFT devices usually require carrier concentrations no higher than 10^16^–10^17^ cm^−3^; however, In_2_O_3_ can be a degenerate semiconductor having a carrier concentration of up to ~10^20^–10^21^ cm^−3^ when doped.

Other than common substitutional doping strategies to monitor the charge carriers, oxygen vacancies have been introduced as the charge carrier providers in oxide semiconductors. To quantify the amount of oxygen vacancies in oxide semiconductors, the environment of oxygen can be analyzed by XPS and their relative amounts compared to metal–oxygen (M-O) bonds and hydroxyl (-OH) bonds are evaluated [7]. The metal–oxygen bond and the oxygen vacancies are of prime importance for the oxide semiconductor monitoring various aspects of the oxide semiconductor. The oxygen vacancy neutral, or ionized form lie in different position in the bandgap. A first criteria for conduction to occur is the Mott criterion which states that $a_{0}\frac{\varepsilon_{r}}{m_{e}^{*}/m_{e}}\sqrt[{3}]{N}>0.26$, where N is the carrier concentration, a_0_ the Bohr radius, $\varepsilon_{r}$ the relative permittivity of the material, and m_e_* the effective electron mass, and m_e_ the electron mass. The equation states the transition from an insulating to a metallic conduction, and the minimum carrier for the transition to occur.

## 2. In_2_O_3_: General Properties

**Figure 1:**
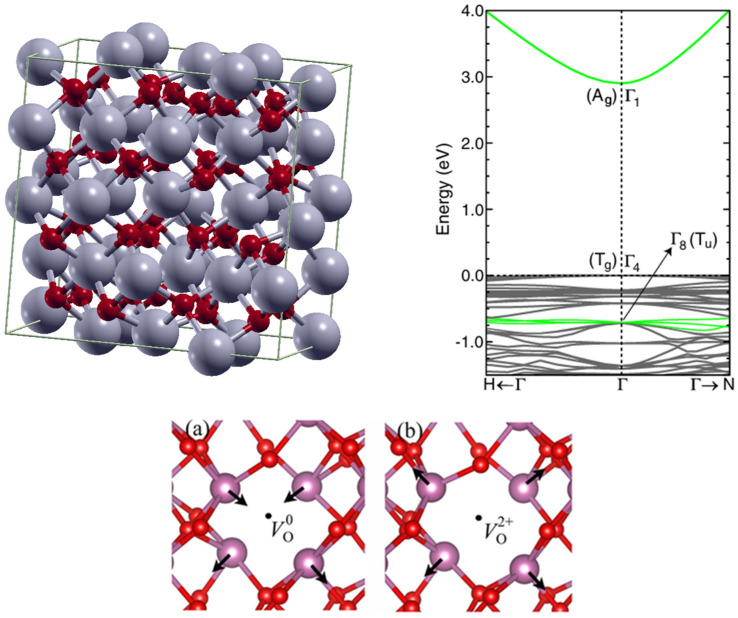
In_2_O_3_ structural and electronic properties. **top left:** In_2_O_3_ crystal structure; **top right:** In_2_O_3_ bandgap evaluation by DFT, reproduced with permission from [9], copyright 2008 by the American Physical Society; **bottom:** impact of neutral and ionized oxygen vacancy on the structure of In_2_O_3_, reproduced with permission from [12], copyright 2019 by the American Physical Society.

In_2_O_3_ is an oxide semiconductor that crystallizes in the bixbyite structure (space group Ia3-); with a lattice constant of 10.12 Å [8] (cf. Figure 1). Each in atom bonds with 6 O atoms, and each O atom bonds with 4 In. Interestingly, the bandgap value has faced various misunderstandings. Owing to the possibility of an indirect bandgap and the Moss–Burnstein band-filling effect, two values have emerged: ~2.9 eV and ~3.7 eV. Density functional theory (DFT) calculations and basic semiconductor physics could have solved the dilemma: the bandgap of 2.9 eV at the Gamma point Γ1–Γ4, even though direct, is parity forbidden (cf. Figure 1). [9] The optical transition occurs by a parity-allowed transition within the valence band and is still at the Gamma point (Γ1–Γ8). The optical bandgap is therefore ~3.7 eV. Let us note that In_2_O_3_ may also crystallize in other forms like the rhombohedral structure or could also be amorphous. DFT calculations showed a bandgap of ~3 eV [10].

As mentioned above, oxygen vacancies are of importance in oxide semiconductors. Neutral oxygen vacancies are reported to lie close to the valence band, while V_o_^+^ or V_o_^2+^ lie closer to the conduction band minimum (CBM) [11]. The effect of a neutral oxygen vacancy and an ionized oxygen vacancy on the structure is shown in Figure 1. Depending on their charge, the oxygen vacancies modify the structure around them. For In_2_O_3_ the V_o_^2+^ demonstrates a stronger repulsion of the various atoms around them than the neutral V_o_ [12].

Oxygen vacancies not only have an impact on the crystallinity and the electronic structures, they also have an impact on the carrier concentration [12]. They are considered to be shallow donors in In_2_O_3_, as in SnO_2_ or ZnO [13]. The formation energy being so low (or even negative) may compensate the acceptor dopants, therefore limiting (i.e., compensating) the p-type possibilities in In_2_O_3_ [12,14]. In fact, the electrons were identified to have originated from surface oxygen vacancies, as the surface oxygen vacancies lie closer to the CBM than their bulk counterparts [15,16,17]. Therefore, to quantify the possibility of having a conductive surface, it is necessary for the surface states to be considered, and the position of the charge neutrality level (CNL) can explain in part the phenomenon [17]. The CNL reveals the position of the change from valence band (or donor)-like to conduction band (or acceptor)-like states at the surface of In_2_O_3_. Whereas in most semiconductors the CNL lies within the bandgap, in In_2_O_3_ the CNL is in the conduction band, at ~Ec + 0.4 eV [8]. In the Fermi level lying below the CNL there is a positive net charge due to unoccupied donor surface states. Therefore, an accumulation of electrons can occur at the surface [18]. The CNL can be estimated by various methods [3,19,20,21,22,23,24]. In the case of a 3D semiconductor with an energy gap <EG> averaged over the Brillouin zone (BZ), the CNL energy is estimated at <EG>/2. Secondarily, by zeroing the Green’s function over the whole Brillouin zone *G(E) = ∫_BZ_∫N*(*E′*)*dE′*/(*E* − *E′*) = 0. Another method consists of calculating the CNL position from the bands calculated by DFT. The CNL value (E_CNL_) can be evaluated through the following equation [25,26]:

$$E_{CNL}=\frac{1}{2N}\sum_{k\epsilon BZ}\left(\frac{1}{N_{C}}\sum_{i}E_{Ci}\left(k\right)+\frac{1}{N_{V}}\sum_{i}E_{Vi}\left(k\right)\right)$$

where N and N_C_ (N_V_) are the number of k points and the number of bands to be considered in the conduction band (valence band).

As a consequence, oxygen vacancies and surface states can explain partially the intrinsic n-type conductivity of In_2_O_3_. Substitutional doping is also a common way to monitor the charge carriers, and Sn has been one of the main dopants in In_2_O_3_ to form ITO. Yet, it was demonstrated that the In_2_O_3_ and Sn-doped In_2_O_3_ have carrier concentration limits [27]. They are determined by the transition of the oxygen vacancy to the neutral state and to the reduction of Sn^4+^ donors to Sn^2+^ electron traps, respectively. The ultimate carrier concentrations achievable by Sn doping and by oxygen vacancies are estimated to be ~1.8 × 10^21^ cm^−3^ and ~6 × 10^20^ cm^−3^. Other possible limiting factors that could also be considered but have a limited impact are the reduction in In (from In^3+^ to In^+,^ or even down to metallic In^0^) and the segregation of the dopant on the surface or grain boundaries.

**Figure 2:**
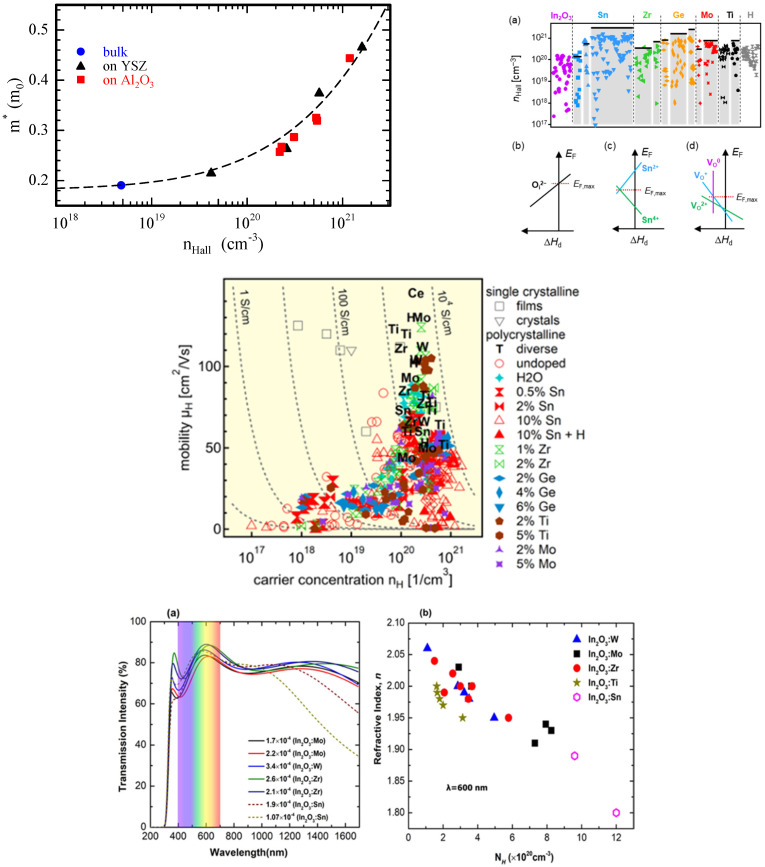
Electrical and optical properties of pristine and doped In_2_O_3_. **Top left**: effective mass as a function of the carrier concentration (reproduced with permission from [30], copyright 2016 by the American Physical Society); **top right**: carrier concentration dependency on the dopant, reproduced with permission from [27], copyright 2024 by the American Physical Society; **middle**: mobility as a function of the carrier concentration with various dopants, figure under CC BY license, reproduced from [28]; **bottom**: optical properties as a function of In_2_O_3_ doping, reproduced with permission from [32], copyright 2021 by the American Physical Society.

Dopants other than Sn, like Zr, Ge, Mo, or Ti, were tried in In_2_O_3_ to increase the carrier concentration. Each dopant could lead to a certain value of carrier concentration, even though carrier concentrations as high as thoise in ITO could not be achieved (cf. Figure 2). In the case of Ti doping, it was further demonstrated that TiO_2_ would form in In_2_O_3_, leaving oxygen vacancies in In_2_O_3_ [28].

Using Mathiesen rule, the mobility can be understood to be limited by polar optical phonon at low carrier concentrations and acoustic deformation potential at higher carrier concentrations. At even higher carrier concentration (>10^20^ cm^−3^) ionized scattering limits the mobility [29]. The effective mass of In_2_O_3_ has been investigated and various values ranging from 0.18 to 0.5 m_e_ were found, [30] with high (low) values for high (low) carrier concentrations. The effective mass is drastically dependent on the carrier concentration, and high carrier concentration can be obtained by substitutional doping (e.g., Sn). This phenomenon has been understood by the non-parabolic nature of the curvature of the conduction band,(cf. Figure 2) and was first expressed by Pisarkiewicz et al. as follows: [31,32]

$$m^{*}=m_{0}^{*}\sqrt{1+2C\frac{\hbar^{2}}{m_{0}^{*}}{\left(3\pi^{2}N\right)}^{2/3}}$$

where *m*_0_^∗^ is the effective electron mass at the conduction band minimum, ℏ the reduced Planck constant, N the carrier concentration, and C a constant (in eV^−1^).

Sn-doped In_2_O_3_ has, therefore, slightly higher effective mass than In_2_O_3_, and has limited transmittance, especially in the IR region. So, to cope with this trade-off of high carrier concentration, low transmittance, and low mobility, other dopants have been investigated. For this purpose, transitional metal (TM) has been introduced in replacement of Sn. Egbo et al. [32] investigated W, Mo, Ti, and Zr doping and showed that Ti-doped In_2_O_3_ and W-doped In_2_O_3_ can have low CB edge effective masses as low as ∼ 0.11−0.14 me. An effective mass of ~0.2 me for carrier concentration in the 1–2 × 10^20^ cm^−3^ range can be obtained. Let us note that the nonparabolicity was verified up to ~5 × 10^20^ cm^−3^, and above that value scattering due to grain boundaries leads to even higher effective masses. The effect on the optical properties is shown in Figure 2.

The role of H is predominant in semiconductors. In amorphous Si, H can suppress dangling bonds [33]. In oxide semiconductors, and especially in In_2_O_3_, it was shown that H can behave as a donor [34,35,36]. When introducing H by various forms like H_2_ or H_2_O during the deposition phase of an amorphous In_2_O_3_, the annealed crystallized In_2_O_3_ films have higher mobilities and carrier concentrations than in the undoped In_2_O_3_ layers. Koida et al. demonstrated that mobility in the 98–130 cm^2^/Vs range can be achieved by suppressing grain boundaries’ defects and neutral impurities. The same group demonstrated the role of transition metal (W, Zr, or Ce) and hydrogen in the mobility of In_2_O_3_. They showed that their introduction in the amorphous phase impacts drastically the crystallized In_2_O_3_ carrier concentration and mobility. The values were even higher than conventional Sn-doped In_2_O_3_ (ITO). They demonstrated that the dopant orbitals lie higher than the Fermi level (E_F_), therefore having no significant impact on the free electrons. The high mobility (µ= e τ/m_e_*, τ being the relaxation time, m_e_* the effective electron mass, e the charge of the electron) could be ascribed to the long relaxation time rather than the small effective mass because of the non-parabolic nature of the conduction band and ionization impurity scattering mechanism [34]. The same group demonstrated the fabrication of 150 nm-thick In_2_O_3_:Ce:H (noted ICO:H) having a mobility of 130 cm^2^/Vs, a carrier concentration of 2.2 × 10^20^ cm^−3^, and an electrical resistivity of 2.13 × 10^−4^ Ohm.cm on PET films [37]. H can also increase the grain lateral growth in solid phase crystallization (SPC) at a low temperature budget of 250 °C [38].

## 3. Introduction to TFTs

In this section, we provide the most important information about TFT parameters. For more details on the TFT working operation the reader is invited to refer to [33,39,40,41,42]. The main parameters to assess a TFT at the initial condition are the threshold voltage Vth (in V), subthreshold swing (SS in V/dec.), and the field effect mobility µ_FE_ (in cm^2^/Vs). In general, Vth is defined as W/L × 10 pA. *SS =* $\frac{\partial V_{GS}}{\partial log(I_{DS})}$, and the field effect mobility is defined as $\mu_{FE}=\frac{\partial I_{DS}}{\partial V_{GS}}\frac{L}{WC_{ox}V_{DS}}$, where W, L, Cox, and V_DS_ are the TFT width, the TFT length, the oxide capacitance, and the applied V_DS_. In large area applications, like active-matrix organic light-emitting diodes (AMOLED), the higher the TFT mobility is, the smaller the SS is and the closer to (positive) 0 V the V_Th_ is, the better. Because of the presence of states within the bandgap, the TFTs are subject to instability. Two instabilities are conventionally tested: negative and positive bias stresses. Negative bias stress (NBS) consists of an applied negative V_GS_ during an amount of time (at least 1 h) and measured transfer I-V curves at specific points of time, whereas positive bias stress (PBS) consists of an applied positive V_GS_ during an amount of time and measured transfer I-V curves at specific points of time. [33,42] To accelerate any process of degradation that may occur, temperature can be added during the stress (the temperature is usually set below 100 °C), and the TFTs are under positive bias temperature stress (PBTS) or negative bias temperature stress (NBTS). Usually, PBS (NBS) leads to charge carrier trapping (untrapping) which results in the positive (negative) shift of the TFT transfer curve. Note that the charge carrier trapping leads to a variation in Vth with stress time, which follows a stretched exponential equation given by ${\Delta V}_{th}\;=V_{th}(\infty )(1-e^{{-(t/\tau )}^{\beta }})$, where $V_{th}(\infty )$ is the Vth shift at infinite stress time, τ a time constant, and β an exponent [43]. But other degradation mechanisms are possible when (for example) a negative shift occurs under PBS, emphasizing the possibility of defect state creation. Specific to oxide-semiconductor-based TFT is also the negative bias illumination stress (NBIS), in which an NBS is applied under light illumination (usually UV). One of the main mechanisms leading to instability under NBIS has been the generation of electrons from ionization of oxygen vacancies, which do not fully recover to their neutral form after the illumination is over [11,44].

## 4. Introduction to Vacuum and Non-Vacuum Processes

As much as possible we have tried to discuss undoped In_2_O_3_ TFT, but in this section some doped In_2_O_3_ TFT may also be introduced when necessary. Full discussion of doped In_2_O_3_ TFT is discussed in another section.

**Figure 3:**
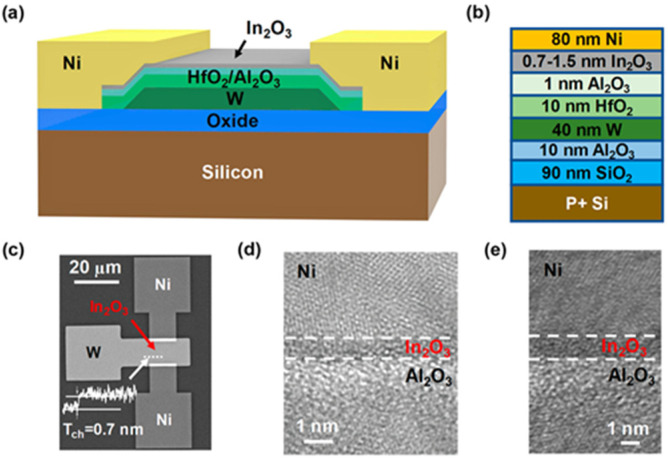
Extremely thin In_2_O_3_ TFT. Reprinted with permission from reference [48]. Copyright 2021 American Chemical Society.

Various processes have been investigated to produce In_2_O_3_ and manufacture TFTs. Among them vacuum processes like sputtering and atomic layer deposition (ALD) have been widely used. Non-vacuum processes like solution process or liquid metal printing have been investigated. Each process has its pros and cons. While the vacuum process has been the process of choice for the development of amorphous oxide (IGZO) TFTs [1,45,46,47], especially by sputtering, the downscaling of In_2_O_3_ allows other techniques to be used. For example, atomic layer deposition (ALD) seems to be a good deposition process technique candidate for In_2_O_3_ layers. Even though the process is rather slow, the technique is suitable for extremely thin and smooth layers. In_2_O_3_ layers as thin as 0.7 nm-thick have been manufactured and used in TFTs (cf. Figure 3) [48]. Also, ALD processed TFTs can have very thin layers and small channel length devices (L_ch_) down to the nm range. Process temperature can be as low as 200 °C, and the mobility of the TFT can be higher than 100 cm^2^/Vs. Solid phase crystallization (SPC)-based TFT can be used to obtain very high-quality poly-crystalline In_2_O_3_ TFTs with a mobility reaching 100 cm^2^/Vs. On the other hand, non-vacuum coatings like spin-coating or spray pyrolysis can be used as alternatives for large devices and for where the thermal budget can be higher than 300 °C. Finally, liquid metal printing can be used at a low temperature as low as RT, with moderate mobilities and small areas, and has proven the development of ~nm-thick layers of various oxide layers. Table 1 summaries all basic pros and cons of the various processes that will be further discussed in the following sections. As mentioned above, one of the aims is the reduction in the channel thickness to obtain 2D-like material behavior. Monitoring the channel layer thickness in TFT also leads to monitoring the number of traps within the bandgap. Nonetheless, a minimum layer thickness λ is required and is given by the equation [33,42].

$$\lambda =\sqrt{\frac{t_{insulator}t_{In2O3}\varepsilon_{In2O3}}{\varepsilon_{insulator}}},\;$$

where t_insulator_, t_In2O3_, ε_insulator_, and ε_In2O3_ are the insulator thickness, the In_2_O_3_ thickness, the dielectric constant of the insulator, and the dielectric constant of In_2_O_3_.

A few research groups could compare vacuum and non-vacuum processes. S. L. Moffitt et al. compared Ga-doped In_2_O_3_ TFTs made by PLD and by combustion process, via spin-coating or spray deposition [49]. PLD-processed layers were revealed to have higher density and carrier mobilities, while the Ga content controlled the carrier concentration in both cases. The combustion process demonstrated also high In-O concentration and low defects. The best mobility (and Gallium %) for the PLD- and spin-coated and sprayed-deposited IGO layers were 42.13 (9.3%), 17.67 (13.9%), and 13.29 cm^2^/Vs (26.2%), respectively.

On the other hand, M. Guo et al. compared ALD, PLD, and solution-processed In_2_O_3_ TFTs [50]. ALD provided the best TFT performances due to higher formation of M-M and M-O bonds, and also due to the formation of flat surfaces. At 350 °C, the mobility, Vth, and thickness for ALD, PLD, and the solution-processed TFT were 82.5 cm^2^/Vs, −5 V, and 4.5 nm; 62.5 cm^2^/Vs, −9.5 V, and 11.7 nm; and 32.1 cm^2^/Vs, 3 V, and 5.8 nm, respectively. Even though PLD-processed TFTs demonstrated the best stability, the passivated ALD-processed TFTs showed a higher stability because of the decrease in -OH groups in the back channel.

### 4.1. Vacuum Process

In this part, we focus on ALD. We discuss the roles of precursors, process temperature, substrates, and thickness. Precursors of indium oxides have an impact on the layer properties [51]. For example, (3-(dimethylamino) propyl)-di methyl indium (DADI) and (N,N-dimethyl butylamine) trimethyl indium (DATI) were compared in a study by H. Y. Lee et al. [52]. Through experimental and theoretical calculations by DFT, they demonstrated that the DATI precursor showed a smaller projection area and reduced steric effects compared to DADI and that this could lead to higher In_2_O_3_ thin-film quality and higher performance of the In_2_O_3_ TFTs. The 400 °C annealed layers made from the DADI and DATI precursors demonstrated similar roughness values, of 0.63 and 0.65 nm, and density values, of 6.57 and 6.76 g/cm^3^. The TFTs made with the DADI and DATI precursors had mobilities of 90.5 and 115.8 cm^2^/Vs, respectively; an SS of 69.2 and 65.6 mV/dec., respectively; and a Vth of −0.22 and −0.12 V, respectively. H.I. Yeom et al. used PEALD with a liquid precursor of Et_2_InN(SiMe_3_)_2_, and showed that the polycrystalline In_2_O_3_ TFT mobility increased from 25 to 39 cm^2^/Vs when the process temperature increased from 200 to 250 °C [53]. J.H. Lee and coworkers fabricated 5 nm-thick In_2_O_3_ layers from the precursor (N,N′-di-tert butylacetimidamido)dimethyllindium and obtained TFTs with mobilities of 55 cm^2^/Vs by adjusting the deposition temperature [54].

Deposition temperature is important and can lead to drastic variations in performance. K S Yoo et al. deposited 10 nm-thick In_2_O_3_ layers and compared the process temperatures of 175, 200, and 225 °C, showing that at 225 °C the highest mobility of ~70 cm^2^/Vs and the best stability against PBS are obtained. The instability was due to electron trapping at the grain boundaries [55]. Applying a high deposition temperature is not necessarily a synonym for higher TFT performances. S.H. Choi et al. showed that an optimum 250 °C deposition temperature could lead to high-performance c-axis aligned crystals In_2_O_3_ TFTs with a mobility of 41.12 cm^2^/Vs, whereas at 300 °C the mobility was degraded down to 16.24 cm^2^/Vs and even to 14.32 cm^2^/Vs at 350 °C because a more polycrystalline material would be formed [56].

The surface on which the In_2_O_3_ is deposited is important, even for bottom gate TFTs. T. Kim et al. showed that having an Al_2_O_3_ layer deposited on top of the HfO_2_ before depositing the In_2_O_3_ can significantly improve device performance. The mobility improved from 40 (without Al_2_O_3_ interlayer) to 148 cm^2^/Vs (with Al_2_O_3_ interlayer). The authors explained the improved performances are due to the decrease in remote Coulomb scattering (RCS) [57]. Z. Chen et al. demonstrated that deposition of the channel layer on SiO_2_, Si_3_N_4_, or Al_2_O_3_ can lead to drastic changes in TFT performances, especially in terms of hysteresis [58]. The TFT deposited on Si_3_N_4_, SiO_2_, and HfO_2_ had a hysteresis of 12.4, 2.1, and 0.49 V, respectively. Their best Al_2_O_3_ passivated TFT had a mobility of 33 cm^2^/Vs.

Thickness control of the In_2_O_3_ is relatively easy with ALD, but a few groups reported a transition from the amorphous phase to the crystalline or even polycrystalline phases. For example, C-E. Oh et al. showed that at a critical thickness of 6 nm a transition from the amorphous phase to the crystalline phase occurs, and by 7.5–8 nm a transition to a polycrystalline structure occurs [59]. They could obtain a mobility of 82.2 cm^2^/Vs. The polycrystalline TFT showed a mobility of 53.3 cm^2^/Vs. The optimized TFT demonstrated V_TH_ shifts of 113 mV under PBS and of 165 mV under PBTS. Another method to crystallize In_2_O_3_ is through a capping layer. Ta or Al have been used and demonstrated mobilities of 101 and 65 cm^2^/Vs at a maximum process temperature of 300 °C, respectively [60]. They induce the crystal growth of preexisting grains in the channel, especially at the capping layer/In_2_O_3_ interface. Ta leads to higher mobility by attracting more oxygen, leading to a more oxygen deficient region in the underlying In_2_O_3_ layer. Thickness control can lead to an amorphous phase In_2_O_3_ with embedded nanocrystals. TFTs with high performances and mobilities of 61.1 cm^2^/Vs were used for flexible applications [61]. Let us note that another group demonstrated that annealing at 275 °C in O_2_ for 1 min is enough to turn a 2.5–3.5 nm-thick amorphous In_2_O_3_ deposited at 250 °C into a polycrystalline structure [62]. Extremely thin layers of In_2_O_3_ as low as 0.5 nm have been implemented into TFTs, even channel lengths (L_ch_) down to 8 nm were manufactured with amorphous In_2_O_3_ [63]. The possibility of such a small TFT is possible because of the CNL being in the CBM, as explained beforehand, which allows a high enough carrier concentration to be available in the channel region [48,62,63]. Because of the high carrier concentration, a V_th_ dependency arises [48]. Also, confinement effects cannot be discarded, which manifest as an increase in the bandgap. Also, the Peide group introduced the trap neutral level (TNL) to explain some of the observed experimental results. For example, TNL could explain trends under PBS or PBTS [64]. More will be discussed below in the IGO section. Also, by thinning down the layer the TNL enters the bandgap, therefore explaining why lower carrier concentrations are available in thinner layers than in thicker layers. The effective mass is also dependent on the thickness [62], and high mobilities can be explained by the theoretically relatively high electron velocity (3.4 × 10^7^ cm/s) being even higher than in Si (1.4 × 10^7^ cm/s). Another advantage of the ALD process is the possibility of fabricating devices with a very high on/off ratio. For example, A. Charnas et al. reported an on/off ratio of 10^17^! [65].

### 4.2. Solution Process

**Figure 4:**
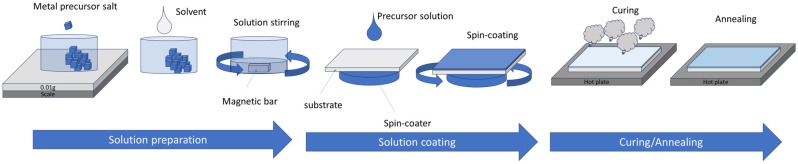
Principle of solution processing with the example of spin-coating. First, the precursor solution is prepared by weighing an amount of metal precursor salt (for example In(NO_3_)_3_. Then the salt is mixed with a solvent (like 2-Me) and left to stir for a few hours. Then the solution is deposited and spin-casted. The remaining liquid is cured to remove the solvent and small particles and form the first M-O-M bonds. Finally, annealing to finish the preparation of the metal oxide semiconductor.

The solution process requires the use of a precursor solution made of at least a solvent and salts which are made of two parts: the metal used in the final oxide semiconductor layer and ligands. The most popular precursor ligand has been nitrate (NO_3_)^−^, as it is further used in the so-called combustion process [66,67]. The most popular solvent has been 2-Methoxyethanol (2-Me). The solution is left to age for a few hours before being spin-casted, cured, and annealed. The curing step is usually at a temperature high enough to evaporate the solvent. At this step, some M-O-M bonds may form. The annealing step leads the M-O-M skeleton to be formed. The basic solution process by spin-coating is shown in Figure 4.

In parallel to the development of the solution process of oxide semiconductors, high-k dielectrics have also been employed, offering a boost in field effect electron mobility [68,69]. One of the goals is to compete with vacuum processes in terms of the process temperature budget. Therefore, many reports have used limited process temperatures down to 300 °C. Indium nitrate mixed into 2-Me has been reported many times. By 200 °C, the mobility hardly reaches 0.1 cm^2^/Vs [66,70,71,72]. Various investigations showed that a process temperature of 300 °C could lead to mobilities of 5–15 cm^2^/Vs [73,74]. At 400 °C, the TFT may also reach a 5–15 cm^2^/Vs, but higher mobilities of 26 cm^2^/Vs can be achieved, for example, by solution engineering [75].

Various strategies have been implemented to boost mobility, like the combustion process. The process uses the precursor for the metal as a source of the oxide metal, the ligand (NO_3_)^−^ as an oxidizer, and a type of fuel like urea or otherwise [66,76,77,78]. The process allows to decrease the activation energy of the reaction involving the creation of the M-O-M skeleton in the layer. Layers can be inactive by conventional solution processing (i.e., mobility in the 10^−4^ cm^2^/Vs range) but can have mobilities of up to 3.37 cm^2^/Vs at 250 °C by the combustion process [66]. Spin-coated In_2_O_3_ being manufactured at as low as 200 °C can lead to mobilities of 0.83 cm^2^/Vs, and by 300 °C, mobilities of 6.57 cm^2^/Vs are possible. The combustion process combined with spray-coating has also been investigated and has demonstrated rather higher results than spin-coating. Mobilities of 15.445 cm^2^/Vs and 1.44 cm^2^/Vs are achievable at 300 °C and 200 °C, respectively. Nonetheless, a large negative Vth at 300 °C (<−15 V) and a large positive Vth at 200 °C (>9 V) were observed [76]. Finally, let us note that careful understanding of the relation between the sprayed droplets on the substrate, the substrate temperature, the nucleation, and the growth of the In_2_O_3_ layer can lead to high mobilities, (30–40 cm^2^/Vs) even without the combustion process [79]. Let us note that the solvent here was not 2-Me but water. J. Yang et al. demonstrated the use of H_2_O_2_ in the solution and IR annealing to produce TFTs with mobilities of 31.7 cm^2^/Vs [80]. Combining solution processing with high-quality ALD-deposited HfO_2_ can lead to mobilities of 19.6 cm^2^/Vs on glass substrates [72].

Previous techniques to improve the solution-processed In_2_O_3_ TFTs involved the use of UV treatments [81,82]. The high energy of UV photons (λ < 300 nm) can be used to efficiently remove carbon-based unwanted residues and can also help to enhance the M-O-M density [81,83]. One of the first reports on UV photo-activated In_2_O_3_ showed In_2_O_3_ TFTs with mobilities of 11 cm^2^/Vs. Even though no external source of heat or annealing are further given, let us note that unintentional heating of ~150 °C could happen during exposure [81]. C.-M. Kang et al. also showed the improvement in TFT with UV-ozone treatment. UV exposure 30 min before annealing at 200 °C lead to a mobility of ~1.25 cm^2^/Vs, whereas the untreated TFT had a mobility of 0.63 cm^2^/Vs [84]. Also, E. Carlos et al. demonstrated the use of inkjet printing on the combustion process and underwent FUV treatment on Al_2_O_3_ gate insulator (GI) to obtain a ~2.8 cm^2^/Vs TFT at a temperature as low as 180 °C [83]. In conjonction with solution engineering (i.e., addition of ammonium nitrate in the solution), deep UV (DUV)-treated layers could have an increase in mobility from 0.21 up to 5.03 cm^2^/Vs [85]. Solution processed-In_2_O_3_ on ZrO_2_ using DUV with a maximum process temperature of 280 °C also demonstrated high performances with a mobility of ∼44.2 cm^2^/Vs. They explained that the quality of their TFT is in part due to the low interface traps [86]. Also, S.K. Shi et al. showed that 2D MoS_2_ doped into an In_2_O_3_ channel layer undergoing UV/O_3_ treatment during the process can lead to stable performance after one month with mobility only decreasing from 2 to 1.8 cm^2^/Vs [87]. R. A. John et al. showed that UV treatment can enhance crystallization and obtain solution-processed TFT with a mobility as high as 30 cm^2^/Vs at a processing temperature as low as 150 °C, while their reference sample annealed at 250 °C only had a mobility of 1 cm^2^/Vs [88]. Chemical impurities were effectively removed and the M-O-M skeleton was effectively formed. Annealing in O_2_/O_3_ was reported to drastically improve the TFT performances [89], also helping to reduce the process temperature. SY Han et al. obtained a mobility of 22.14 cm^2^/Vs at 300 °C and a mobility of 0.85 cm^2^/Vs when annealing at 200 °C. Compared to their air-annealed TFT, the O_2_/O_3_-annealed In_2_O_3_ layer showed indium atoms with six oxygens as their nearest-neighbor and high density of oxygen vacancies, whereas the air-annealed films had less coordinated In and more hydroxyl groups. Solution-processed In_2_O_3_ TFTs with ALD Al_2_O_3_ on top of solution-processed Ce-doped Al_2_O_3_ (CeAlO) showed significant improvement over TFTs without CeAlO, with their mobility increasing from 9.11 to 26.86 cm^2^/Vs for Ce:Al = 3:7. The stability was also drastically increased, with a reduction in Vth shift under PBS from 0.31 V down to 0.05 V for the TFT without a CeAlO layer and with a CeAlO layer (Ce:Al = 3:7), respectively [90]. Note that the maximum process temperature was 450 °C.

Therefore, to obtain higher performances in the solution process, either new solution engineering, new coating techniques, or higher annealing temperatures could be required. The example of using Cl ligands will be introduced in the IGO section.

### 4.3. Liquid Metal Printing

**Figure 5:**
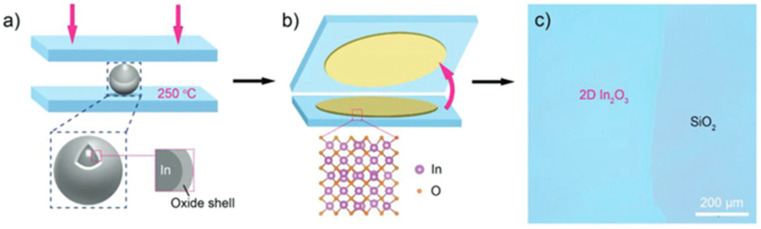
Principle of liquid metal printing applied to In_2_O_3_. Figure under CC BY license, reproduced from [92].

Recently, liquid metal printing has attracted attention because of its ease in manufacturing 2D oxide semiconductors at a relatively low process temperature. The process consists of using a pure or a combined material (usually near eutectic composition) [91] and squeezing it under monitored pressure between a TFT backplane and a counter substrate. By Cabrera-Mott (CM) oxidation, an oxide thin layer will form at the surface of the material and will be printed on the substrates. The concept is shown below in Figure 5. The process is eventually repeated to obtain thicker layers. One layer is usually in the nm range (<2 nm). For example, In_2_O_3_ was manufactured by liquid metal printing. A 2 nm-thick layer could have large grains of 16.2 nm, and this has led to a TFT mobility of 96 cm^2^/Vs [92]. Another report showed that increasing from one layer to two monolayers could drastically increase the mobility from 2.6 to 67.1 cm^2^/Vs at a maximum process temperature of 250 °C [93].

The liquid metal printing method is used when limited to a small area. For larger areas, continuous liquid metal printing (CMLP) has been introduced and proves that printing a few meters per minute is possible. Following this method, IGO was deposited and mobilities of ~10 cm^2^/Vs were achieved [94]. Other methods and more about liquid metal deposition can be found elsewhere [91].

## 5. Material Optimization

Material optimization regroups all strategies to improve the semiconductor characteristics like TFT stability, carrier concentration, mobility, etc. Doping is one strategy to reach one or more of these improvements. Doping not only improves carrier concentration but also reduces defects. Indeed, as previously mentioned, oxygen vacancies play a major role in the number of electron density. TFTs require charge carriers of up to ~10^16^–10^17^ cm^−3^, so the 10^20^ cm^−3^ carriers that could be available in In_2_O_3_ are too many. To understand the ability of a dopant to reduce the number of charge carriers, two aspects have been introduced: the standard electrode potential (SEP) [95,96,97] and the metal–oxygen dissociation energy [98]. A dopant atom having a lower SEP than that of In would emphasize the dopant’s stronger ability to make a metal–oxygen bond. Also, a dopant strong bond energy would also mean that creating an oxygen vacancy is more difficult to form than from the In-O bond. Another aspect is the size of the dopant, that should be of similar size to In^3+^ in order to not perturbate the crystal and the lattice constants. Dopants can also, as discussed below, lead to better initial characteristics and higher stability under various stresses. We will now introduce various dopants. Here, we will not introduce doping with more than one cation, so that IZTO and IGZO are left out of this review. Table 2 below summarizes the various dopants introduced in the following sections and their uses in TFTs.

### 5.1. Various Dopings

Many dopants have been used to replace the cations but few tries to substitute the oxygen anion have been reported. S introduction was reported to have a significant improvement on the TFT characteristics, as S-O has a higher bond energy of ~522 kJ/mol compared to the In-O bond energy of 320 kJ/mol. C-axis oriented ~2.7 nm-thick In_2_O_3_ TFTs without S had a high mobility of ~33 cm^2^/Vs but a small on/off ratio of 2.3 × 10^5^, while the 1% S-doped In_2_O_3_ lead to a mobility of ~22 cm^2^/Vs and an on/off ratio of 1.2 × 10^7^ [99].

Li and B and Sb have been investigated as potential dopants but not many reports have been published. A solution-processed 13.5% Li-doped In_2_O_3_ TFT had a mobility of 60 cm^2^/Vs was compared to an undoped In_2_O_3_ TFT with a mobility of ~20 cm^2^/Vs [100]. At a max temperature of 350 °C, 5 and 10% boron-doped In_2_O_3_ showed a mobility of 11.18 ± 0.6, 7.98 ± 0.63 cm^2^/Vs, whereas the undoped one had ~28 cm^2^/Vs but with an improved on/off ratio from ~7 × 10^3^ to 1.04 and 2.84 × 10^6^ and a Vth from −9.49 ± 0.96 to −1.88 ± 0.16 and 3.96 ± 0.15 V [101]. Another study showed a solution-processed 6% boron-doped In_2_O_3_ on boron-doped Al_2_O_3_ having mobility of ~11 cm^2^/Vs [102]. They demonstrated theoretically and experimentally that the boron allowed the In_2_O_3_ to become amorphous and decreased the carrier concentration. On the other hand, boron has also been used in SPC In_2_O_3_ revealing its potential to reduce crystal defects and in In_2_O_3_ to achieve mobilities of ~87 cm^2^/Vs [103]. Through metal liquid deposition very thin Sb-doped In_2_O_3_ were used in TFTs. The 2–3 nm-thick channel layers lead to a high mobility of ~40 cm^2^/Vs, compared to the undoped In_2_O_3_ having a mobility of ~5 cm^2^/Vs. One of the reasons of the improvement is the higher sheet carrier concentration that increased from 5.3 × 10^12^ to 1.6 × 10^13^ cm^−2^ [104].

The 290 °C manufactured solution-processed 1% Hf-doped In_2_O_3_ TFT lead to mobilities of ~2.6 cm^2^/Vs relatively lower than the undoped In_2_O_3_ TFT with a mobility of 8 cm^2^/Vs but the doped In_2_O_3_ TFT also demonstrated a lower SS [105]. On the other hand, 250 °C manufactured ALD-deposited Hf-doped In_2_O_3_ TFT demonstrated mobilities of 18.65 cm^2^/Vs. The annealing temperature controlled effectively the TFT performances by modulating the concentration of oxygen vacancies, [106] while at 300 °C the same group demonstrated an optimal composition of In_2_O_3_:HfO_2_ = 10:1 leading to a mobility of 13.4 cm^2^/Vs. They also demonstrated by DFT calculation that the decrease in carrier concentrations is due to the creation of gap states [107].

Mo was demonstrated to rapidly degrade the performances of the TFT in terms of mobility. Tarsoly et al. showed that 250 °C annealed solution-processed Mo-doped In_2_O_3_ demonstrated a decrease in the mobility and V_Th_ from 3.34 down to 0.20 cm^2^/Vs and from 10.6 down to 3.9 V by increasing the Mo content of only 0.67 mol% in the film; but the TFTs demonstrated higher stability under stresses [108]. The same group showed, by changing the solvent from hydrogen peroxide to DI water, higher performances can be obtained in terms of mobility and stability under PBS/NBS and stability overtime [109,110,111].

Let us note that in all these previous examples, a limited amount of dopant is enough to either amorphize the In_2_O_3_ layer or to reduce the carrier concentration.

Y. Zhou et al. reported in 2012 a way to monitor the work function of electrodes was by coating with PEI or PEIE [112]. From this work, the concept of polymer doping emerged. PEI and PEIE have both been introduced into solution-processed In_2_O_3_ solutions. W. Huang et al. worked on PEI and PVA doping [113,114]. They showed an increase in mobility from 4.18 to ~8.37 cm^2^/Vs with 1% doping PEI, while PVA doping drastically decreased the mobility but increased the on/off ratio [114]. The same group showed that the nitrogen content in the polymer can have a drastic impact on the TFT performances [115]. Authors studied the effect polymers: In_2_O_3_ blends using PEI, PEIE, PVP, PAA, and PVP-NH_2_, and demonstrated the relationship between N content and TFT behavior. Polymers have different effects on the crystallinity of the blend. The authors reported a mobility of 31.24 ± 0.41 cm^2^/Vs, using F:Al_2_O_3_ as a GI for the In_2_O_3_:PEI blends. The same group also demonstrated a 1% PEI-doped In_2_O_3_ TFT with a ZrO_2_ insulator with a mobility of ~30 cm^2^/Vs [116]. On the other hand, M. Divy et al. showed that the addition of ethyl acetate (EC) into an In_2_O_3_ precursor solution can easily lead to a high-performance TFT, with the mobility reaching 40–45 cm^2^/Vs [117], by allowing the layer to crystallize when the undoped layer is amorphous. The annealing temperature was 275 °C. Even though they are not polymers, single-wall carbo nanotubes (SW-CNT) were also doped into In_2_O_3_ precursor solutions [118]. The doped (undoped) layer had a mobility of 1.61 (0.59) and an SS of 0.48 (1.1.7 V/dec). By trapping some electrons, the CNT helped reduce the off current and enhanced the stability under NBS.

**Figure 6:**
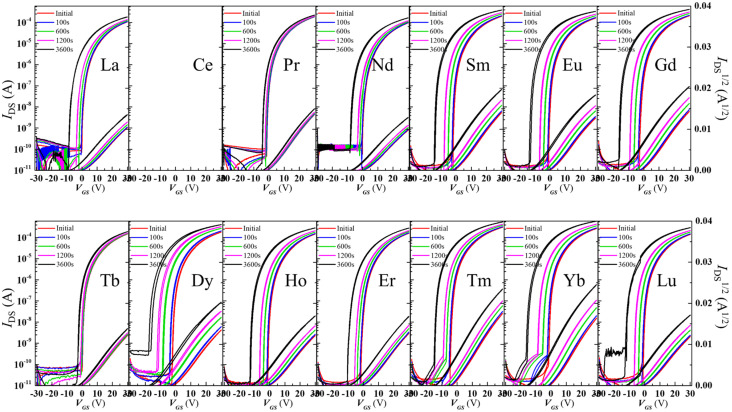
Influence of the Ln dopant on the NBIS behavior of the In_2_O_3_ TFT. (Figure under CC BY license, reproduced from [119]).

Lanthanides have been investigated as dopants in In_2_O_3_ to improve performance. P. He et al. [119]. fabricated and compared 300 °C annealed solution-processed 5% doped lanthanide TFTs. Interestingly the Ce-doped showed no TFT behavior at that concentration. Other lanthanide-doped In_2_O_3_ TFT revealed a drastic drop in mobility compared to the undoped In_2_O_3_ TFT, from 17.1 down to 3–6 cm^2^/Vs, but offered a positively shifted Vth from −13.3 to ~0 V. More importantly the Tb- and Pr-doped In_2_O_3_ showed superior stability against NBIS, and the reason was attributed to absorption of the incident light to the charge transfer transition and to downconversion to the non-radiative transition. Figure 6 shows the effect of lanthanide dopant on NBIS. Sputtered Tb-doped In_2_O_3_ was used for improvement of stability under NBIS by L. Lan et al. [120]. Doping does not significantly modify the initial parameters of the TFT: undoped In_2_O_3_ had a mobility of 51 cm^2^/Vs while Tb 5% doped In_2_O_3_ demonstrated a mobility of 45 cm^2^/Vs. The improvement in V_th_, a shift under NBIS from −11 V down to −3.9 V, was attributed to a decreased density of photo-induced charge carriers in the doped layer than in the undoped In_2_O_3_.

H. Du et al. reported on 500 °C annealed solution-processed La-doped In_2_O_3_ TFTs and demonstrated an optimum doping of 5% to reach a mobility of 14.22 cm^2^/Vs, a V_TH_ of 2.16 V, an SS of 0.84 V/dec, and an on/off ratio of 10^5^ [121]. Z. Wang et al. showed that 400 °C annealed 1% doped Yb In_2_O_3_ lead to the best TFT performance with a mobility of 13.32 cm^2^/Vs, a Vth = 0.11 V, an SS of 0.38 V/dec, and with TFTs that were five times more stable than the undoped In_2_O_3_ TFTs. Similarly to La, Yb was reported to reduce defect density [122]. On the other hand, J Smith et al. conducted theoretical and experimental analysis of Sc-, La-, and Y-doped In_2_O_3_ and applied them to TFTs [123]. The ionic size had an effect on the localization of states: for Sc the states were below, for Y the states were below and at, and for Y the states were below and above the Fermi level. Also, depending on the process temperature and the dopant concentration, they could observe that the TFT conduction mechanism could be governed either by the trap limited conduction (TLC) or by the percolation mechanism (PC).

W can be a good dopant as it can provide more electrons than In and has a stronger bonding dissociation energy. Also, DFT calculations revealed that 1% W-doped amorphous InO_x_ had a higher position of the Fermi level but with the creation of states within the bandgap. Therefore, there should be a trade-off between performance and stability [124]. Mobilities of approximately 30 cm^2^/Vs have been achieved multiple times [125,126,127]. Co-sputtered IWO TFT had a higher performance (mobility ~60 cm^2^/Vs), higher stability under NBS and PBS, and higher uniformity than TFTs made from a sputtered IWO target. The reason was the non-uniformity of W in the sputtered layer from a single target source [128]. Chosen for its high oxygen bond dissociation energy, 0, 1, 2, and 4% W-doped In_2_O_3_ demonstrated mobilities of 76, 41, 12, and 3 cm^2^/Vs, respectively. Also, the TFTs showed high stability. Even scaling down the channel layer length to 60 nm lead to stable TFTs [129]. Ruan et al. demonstrated that the use of supercritical-phase carbon dioxide (SCCO_2_) and H_2_O_2_ as a co-solvent for vacuum processed W-doped In_2_O_3_ can drastically improve performance and lead to a TFT mobility of ~100 cm^2^/Vs with rather good stability under NBS and PBS [130]. The SCCO_2_ and H_2_O_2_ effectively passivated the oxygen vacancies and defects.

Hydrogen incorporation, as mentioned previously, can lead to higher mobilities. Incorporation can be done directly (during the deposition of the In_2_O_3_ layer) or indirectly like during the deposition of another layer (the passivation or the gate insulator for example). Y. Magari et al. demonstrated the importance of H doping while In_2_O_3_ is in the amorphous phase. An annealed SPC-crystallized In_2_O_3_:H layer would have a controlled carrier concentration, large grains (~140 nm), and the TFTs would have mobility of 139.2 cm^2^/Vs, a Vth of 0.2 V, and an SS of 0.19 V/dec [131]. By the control of ALD supercyles, H. Y. Kim et al. monitored the introduction content of H in the gate insulator of a top gate TFT [132]. They demonstrated the introduction of H into the underneath In_2_O_3_ layer by SIMS and elastic recoil detection (ERD). The optimized TFT had a mobility of 159 cm^2^/Vs and high stability against current stress. H passivated defects but also acted as a shallow donor as interstitials.

Controlling the deposition and the interfaces is important. S-H Chun et al. showed that the incorporation of an Al_2_O_3_ layer before the deposition of In_2_O_3_ in a top gate TFT can lead to substantial improvement of the TFT mobility, reaching 223 cm^2^/Vs for the polycrystalline In_2_O_3_ and 25.9 cm^2^/Vs for the amorphous In_2_O_3_, which are substantially higher than the mobilities of 56.9 and 10.4 cm^2^/Vs obtained for the TFTs fabricated without the layer. The performances highly depended on the thickness and annealing of the layer [133]. The incorporation of H_2_ during sputtering allowed P. Hu et al. to reach mobilities of 47.8 cm^2^/Vs, whereas without H_2_ the TFT had a mobility of 37.8 cm^2^/Vs. The decrease in the defect states was the main reason for this improvement [134].

### 5.2. ITO

There has been a regain of interest in ITO, not as a transparent conducting oxide (TCO) but as a possible channel layer for TFT, because of its scaling possibilities and its ability to be used for radio frequency (RF) applications. 4 nm-thick ITO with HfLaO gate insulators [135] were demonstrated to produce very high-performance TFT with a low SS of 66 mV/dec. and a high on/off ratio (over 10^9^). Their 40 nm-long TFT demonstrated a cutoff frequency f_T_ of 10 GHz and a maximum operating frequency f_max_ of 12 GHz. Also, because of the low electron velocity (~3 × 10^5^ cm/s), the thinner layers showed a better RF response than their thicker counterparts.

Hu et al. demonstrated a flexible scaled ITO TFT channel length as low as 15 nm with a high cutoff frequency of 11.8 GHz and an operating frequency of 15 GHz, robust to 10,000 bending cycles [136].

One way to understand the performance of the ITO TFT is to use the CNL concept [26]. The CNL in In_2_O_3_ and in SnO_2_ were calculated [25] to be 0.34 eV and 0.24 eV above the CBM, respectively. The CBM of SnO_2_ being above that of In_2_O_3_, electrons would flow in In_2_O_3_, while SnO_2_ would create an energy barrier, so increasing the content of Sn would decrease the electron mobility. So, a maximum mobility of 28 cm^2^/Vs for an In:Sn = 9:1 ratio was obtained for a scaled TFT with L_ch_ of 60 nm and ITO thickness of 2.1 nm. Sn was further confirmed to inhibit crystallization. A 23% Sn-doped In_2_O_3_ leads to the high 52.7 cm^2^/Vs and a high on/off ratio of 10^9^ [137]. The authors showed a small Vth variation in −0.42 V under 85 °C NBTS and a small Vth variation of 0.017 V over 4 ks PBTS. Increasing the annealing temperature from 150 to 250 °C can enhance the mobility of sputtered ITO TFT from 70.53 up to 100.15 cm^2^/Vs [138]. The TFTs worked in depletion mode with a Vth of −6.78 and −14.51 V, respectively. Passivation had a drastic positive effect on ITO TFTs. Al_2_O_3_ was reported to slightly decrease the mobility from ~44 (for the unpassivated TFT) down to ~34 cm^2^/Vs, but reached an almost 0 hysteresis and a −0.13 V (1.7 V) Vth shift under NBS (PBS) [139].

The liquid metal printing process has been tried for the manufacture of 2D ITO TFT. Y Tang et al. demonstrated a 200 °C processed 1.9 nm-thick liquid metal manufactured ITO layer leading to a TFT reaching a mobility of 27 cm^2^/Vs, and they also showed high-gain inverters [140]. On the other hand, Minh Nhut Le et al. fabricated a bottom gate double channel made of ITO with Sn content of 20% below an ITO layer with a high Sn content 40%. They could obtain mobilities of ~24 cm^2^/Vs for a process temperature below 200 °C [141]. Pre- and post-annealing could help increase the ITO TFT performance as described by G. Gao et al. By pre-annealing at 200 °C and moderate post-annealing at 80 °C, the TFT worked in enhancement mode with a Vth of 6.39 V and demonstrated a mobility of 34.8 cm^2^/Vs [142]. Vacuum annealing has a benefit effect on the performance of sputtered ITO TFT, leading to a mobility of 43.6 cm^2^/Vs and an on/off ratio of 1.26 × 10^7^ [143]. Air annealing could lead to mobilities reaching 86.5 cm^2^/Vs and an SS of 80.4 mV/dec. The authors reported a negative shift in Vth that should result from the generation of electrons from defect states [144].

With O_2_ plasma treatment, S. P. Jeon et al. [145] demonstrated the possibility to control the Vth of the ITO TFT by controlling the carrier concentration. By increasing the power from 100 to 200 W, Vth shifted from −25.81 up to −4.91 V, with a modification in mobility from 21.93 to 8.54 cm^2^/Vs. The reduction in carrier concentration was from 4.67 × 10^20^ to 1.39 × 10^16^ cm^−3^.

Also, W-doped ITO TFT were fabricated, where In was fixed but W substituted some of Sn. W can effectively tune the properties of ITO by shifting the Vth when W:Sn increases from 0:10 to 5:5. For the 5:5 ratio, even though V_on_~0 V, the mobility is ~5 cm^2^/Vs. For 9:1, the mobility is ~10–15 cm^2^/Vs, when the V_on_ is ~0 V. The TFT was further used for proximity detection applications [146].

Analysis of 1/f noise is a powerful tool for the analysis of TFT properties. For example, C. Gu et al. demonstrated that noise in ITO can be attributed to the fluctuation in carrier number, which, as their analysis shows, results in part from the traps [147].

Finally, Q. Li and coworkers tested the effect of various contact source/drain. They emphasize that between Ti, Mo, and Al, Al provides the lowest contact resistance leading to a mobility of 26.45 cm^2^/Vs, and a relative high NBIS stability with a variation in Vth below 1 V [148].

### 5.3. IGO

Gallium-doped In_2_O_3_ has also gained attention recently. High mobility, >30 cm^2^/Vs by the solution process and >70 cm^2^/Vs by the vacuum process, with V_on_ ~0 V have been achieved. Ga doping offers a way to obtain high-performance TFT without using the depletion mode as is usually the case for In_2_O_3_ TFTs. We will discuss the various properties of IGO TFTs reached.

**Figure 7:**
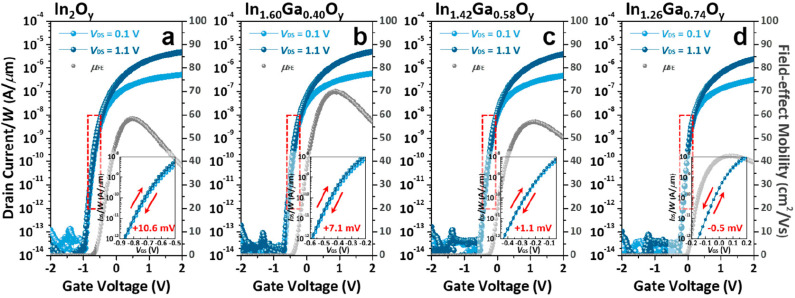
Transfer curves of IGO TFT with various Ga contents. Reprinted with permission from reference [150]. Copyright 2020 American Chemical Society.

First, Ga doping content influences the crystallinity of the material, and crystallization temperature depends on the Ga content. This trend has been observed by various research group using various deposition techniques [149]. Moffitt et al. showed that In_2_O_3_ could crystallize at 125 °C, whereas for 8% doped In_2_O_3_ the crystallization temperature increased to 250 °C, and 51% Ga-doped In_2_O_3_ required 425 °C. The same group showed that, as discussed in a previous section, PLD, combustion-spin coating-processed or combustion spray pyrolyzed IGO have different crystallization temperatures, depending on the Ga content, but also the deposition method [49]. The TFTs demonstrated a decrease in mobility with Ga content, introducing traps and decreasing the carrier concentration, but also modifying the in In-O-In skeleton. J S Hur and coworkers [150] showed that the bandgap value of IGO depends linearly on the content of the doping content. Annealing at 400 °C, the authors observed at 20% a large grain sized IGO, and an amorphous IGO for Ga > 29%. They also showed that the introduction of 20% of Ga leads to an optimum TFT mobility reaching 71.27 cm^2^/Vs, while the amorphous 29% doped IGO TFT had a mobility of 41.21 cm^2^/Vs. Examples of IGO TFT performances are shown below in Figure 7. Annealing has an impact on the performance of the TFT. A small annealing temperature of 220 °C can be enough to obtain a mobility of 35 cm^2^/Vs, whereas increasing the annealing temperature could lead to defect formations [151].

Rabbi et al. showed the crystallization temperature to be 330, 350, and 415 °C for Ga = 20, 30, and 50%, respectively. Fabricated by spray pyrolysis, they obtained 30% doped polycrystalline IGO at a process temperature of 370 °C, and obtained a mobility of 43.73 cm^2^/Vs. They showed high stability against NBS/PBS and used the TFTs in shift registers. The same group reported a 50% doped amorphous IGO TFT with a mobility of ~30 cm^2^/Vs on a flexible substrate, having a small Vth shift (below 0.3 V) under PBTI [152]. H J Yand et al. showed an amorphous IGO for Ga > 34% when processed at 400 °C. A 37% Ga-doped with a maximum process temperature of 700 °C showed a mobility of 60.7 cm^2^/Vs [153].

**Figure 8:**
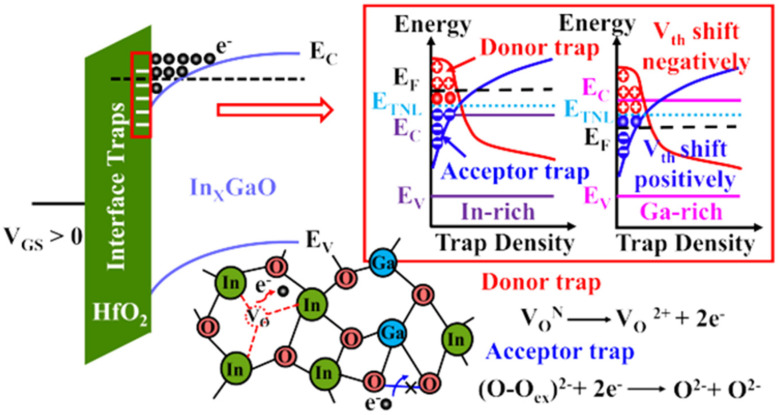
Principle of TNL in InGaO, 1557-9646 © 2025 IEEE. All rights reserved, including rights for text and data mining and training of artificial intelligence and similar technologies. Personal use is permitted, but republication/redistribution requires IEEE permission. See https://www.ieee.org/publications/rights/index.html (accessed on 13 September 2025) for more information, reprinted with permission from [154]. Zhang, J.; Zhou, C.; Dou, H.; Zhang, Z.; Lin, Z.; Xu, K.; Zhang, X.; Wang, H.; Zhu, H.; Yang, W.; Ye, P Effects of Gallium on Electron Transport and Bias Stability in Ultrascaled Amorphous InGaO Transistors IEEE Trans. Elec. Dev., **2025**, *72*, 4156–4162. 10.1109/TED.2025.3583705.

J Zhang et al. reported the stability of IGO TFT as a function of Ga content with a small channel length (down to 60 nm) [154]. They revealed that rich In IGO had a negative shift under PBS, while increasing the Ga content up to a Ga rich IGO TFT showed a positive Vth. The authors proposed to consider the trap neutrality level (TNL), and therefore states near/at the interface (cf. Figure 8). At rich In content, the oxygen vacancies behaving as donor-like states are ionized because of the high applied electric field. At rich Ga content, the material has stronger Ga-O bonds being able to trap electrons. The effect of donor states providing electrons during PBS was reported many a times, for example, with an IGO of In:Ga = 86:14 and their different behaviors in different atmospheres [155].

On the other hand, K Hu et al. suggested that Ga content and defect energy states in the insulator can directly explain the PBS behavior. The higher Ga content would lead to the narrowing of the bandgap, thus separating the electrons from the IGO conduction band further from the insulator trap site energy, which was confirmed by the variation in activation energies in the TFT [156]. Offset TFTs have been used to increase TFT performance. For example, Rabbi et al. demonstrated sputtered IGO TFT having a mobility of 85 cm^2^/Vs with a ΔV_th_ of only 0.1 V under PBTI [157]. J. Hao et al. used offset TFTs on IGO TFTs to obtain a mobility of 35 cm^2^/Vs for use under high voltage with very high on current of 794 µA [158]. They obtained a breakdown voltage of 638 V and Baliga’s Figure of Merit (BFOM) of 2.4 MW/cm^2^.

Various reports have shown the IGO layer deposited as In_2_O_3_/Ga_2_O_3_ stack(s). By ALD, J S Hur et al. fabricated from 1 to 18 stacks, and by keeping the total thickness at 8 nm they showed that 1 stack lead to crystalline In_2_O_3_ with a highest mobility of 94.1 cm^2^/Vs, while the 18-stack IGO layer lead to an amorphous layer with a mobility of 58.1 cm^2^/Vs [159]. They also demonstrated that they obtained a quasi 2D electron gas (2DEG) layer in the In_2_O_3_ layer as obtained from CV measurements, using the following equations to find the electron carrier concentration as a function of the channel depth:

$$N=\frac{-2}{q\varepsilon_{In2O3}\varepsilon_{0}\frac{d\frac{1}{{C\left(V\right)}^{2}}}{dV}},\;x\left(V\right)=\varepsilon_{In2O3}\varepsilon_{0}\left(\frac{1}{C\left(V\right)}-\frac{1}{Cox}\right)$$

where q is the charge of the electron, $\varepsilon_{In2O3},\;$ is the permittivity of In_2_O_3_, $\varepsilon_{0}$ is the permittivity of vacuum, C(V) is the measured capacitance, Cox the oxide capacitance, and x the channel depth.

Let us note the use of the continuous liquid metal layer printing (CLMP) deposition strategy to fabricate stacks. Andrew et al. fabricated In_2_O_3_, In_2_O_3_/Ga_2_O_3_, and obtained 2D TFT with mobilities of 12.3, 13.5 cm^2^/Vs, an improved SS from 1 down to 0.23 V/dec., and a positive shift in Vth from −35 up to −2.3 V. Let us note that the deposition method is ultra-fast, requiring less than 10 s [160]. A 3 nm-thick In_2_O_3_ capped with Ga_2_O_3_ could have a mobility of 9.3 cm^2^/Vs as reported by J. Zhu et al. [161]. Another achievement with ALD-manufactured IGO TFTs (In:Ga = 8:2) is the possibility to reduce strong DIBL effects in scaled TFTs. The authors reported highly oriented IGO TFTs with a DIBL of ~17.5 mV/V and a mobility of 81.9 cm^2^/Vs, whereas randomly crystallized IGO TFTs had stronger DIBL values of ~197 mV/V and a mobility of 46.4 cm^2^/Vs [162].

The deposition conditions are important to consider. For example, introduction of O_2_ during the deposition of IGO induces crystallization, as reported by H J Park et al. [163]. Because of a good crystalline structure, a P_O2_ of 10% leads to an optimum condition of IGO TFT with a mobility of 56 cm^2^/Vs and a Vth of ~0.1 V. Higher P_O2_ leads to smaller grains.

Other than the control of the crystal phase, other dopants have been introduced to obtain other properties. J.B. Bae et al. incorporated La in replacement of Ga with In:(Ga + La) = 7:3. They showed that 3% La doping enhances stability and mobility (obtaining mobility of 34.84 cm^2^/Vs), whereas higher doping concentration degrades the TFT performances [164]. Pr was doped into IGO by Y. Zhu et al. to improve the NBITS stability and light effect. They showed that up to a limited doping amount of 6.12%, the mobility decreases from ~19 down to 15 cm^2^/Vs. The Vth shift under NBITS was significantly decreased, from more than −15 V to less than −5 V for no Pr doping and 6.12% doping, respectively [165].

Without any compensation element, IGO could be used as a UV detector, as reported by W L Hu and coworkers. With a mobility of 2.66 cm^2^/Vs their TFTs had a rejection ratio higher than 10^5^, and a responsivity of 5.012 A/W [166]. MJ Kim et al. demonstrated the role of the substrate on the formation of highly oriented In_2_O_3_ and IGO layers. An underlying Al_2_O_3_/ZnO induced high quality film growth and could lead to mobilities of ~95 cm^2^/Vs [167]. Other achievements related to a double channel layer will be discussed below in the manuscript.

## 6. Device Optimization

In this section, we will discuss strategies to improve the static performances (mobility, SS, Vth) and stability under stress. We will first discuss process effects, the roles of plasma treatments, and how to choose the source and drain electrodes. Then we will discuss dual gate structures, interactions with organic materials, and strategies to effectively passivate the channel layer form the environment.

### 6.1. Process Effects

Partial pressures of H_2_O, power, and other parameters during the deposition of In_2_O_3_ are important to obtain high mobility In_2_O_3_. J. Nomoto and coworkers showed the procedure to obtain mobilities of 100 cm^2^/Vs by sputtering [36]. Gases used during deposition in the vacuum process, like N_2_, have been reported by W. Pan et al. to monitor the In_2_O_3_ TFT mobility. Their optimized TFT showed a mobility of 24.96 cm^2^/Vs and demonstrated a PBS and NBS Vth shift of 10.33 and −1.42 V, respectively [168]. H. Sadahiru et al. used In(OH)_3_ targets for PLD deposition to manufacture TFTs with mobilities of 90 cm^2^/Vs. The TFTs had a small Vth shift under PBS and NBS of 0.51 and 0.17 V, respectively [169]. The advantage was to obtain very large grains of ~2 µm. Let us note that even though MBE has not been widely used for the fabrication of In_2_O_3_ TFTs, Hensling et al. obtained a Hall mobility of 52.9 cm^2^/Vs, and a field-effect mobility of 19.1 cm^2^/Vs [170]. J Zhang et al. showed that pre- and post-annealing with controlling the relative humidity (RH) can drastically induce higher performances in solution-processed In_2_O_3_ TFTs, obtaining mobilities of up to 17 cm^2^/Vs [171]. N. Xiao et al. also showed the importance of the annealing atmosphere, showing that annealing in O_2_ can lead to enhancement mode devices, whereas annealing in N_2_ leads to depletion mode devices [172]. J Wang et al. demonstrated the influence of the annealing atmosphere and the thickness on the TFT performances [173]. N_2_ annealing can lead to the formation of more V_O_, whereas O_3_ annealing can supply oxygen in the layer. They obtained a mobility of 53 cm^2^/Vs and a Vth shift under PBS and NBS of 0.23 and −0.53 V, respectively. On the other hand, C S Huang et al. showed that RTA in an O_2_ environment at 800 °C can lead to a reduced number of defect states and less degradation during annealing in forming gas and obtained a mobility of 5 cm^2^/Vs [174].

### 6.2. Plasma Treatments

Plasma treatment is an effective way to enhance TFT parameters. Their optimization can induce a decrease in defect states. O_2_, CF_4_, and N_2_O plasma treatments are the most employed. O_2_ plasma treatment can lead to the enhancement of stability under PBS/NBS, but a power too high can have the countereffect, creating gap states and negatively impacting the stability [175]. A simple way to detect the defects is optically, by analyzing the Urbach tail below the bandgap. As explained by N Xiao et al., [176] the O_2_ plasma treatment employed before passivation can decrease trap states and ensure a significant boost in mobility from 31.6 to 128.3 cm^2^/Vs. The O_2_ plasma treatment on top of the In_2_O_3_ channel region can reduce defects introduced during the fabrication process, control the Vth, reduce the SS, and replace the annealing step that could be too high to consider further implementation in back-end-of-the-line (BEOL) applications [177]. Finally, let us note that O_2_ plasma treatment was also effectively used to a-IGO to lead to crystallize IGO at an annealing temperature of 350 °C [178]. The authors reported a mobility of 43.2 cm^2^/Vs. Coupled with UV treatment R, Tseng et al. [179] reported the ability to control the Vth efficiently. As others have, they used the Drude model to evaluate the carrier concentration n_2D_ for their 2 nm-thick In_2_O_3_ layer following the equation.

$$n_{2D}=\frac{I_{DS}L}{qWV_{DS}\mu_{FE}},\;\mathrm{evaluated}\mathrm{at}V_{\mathrm{GS}}=0$$

Their 2D carrier concentration was higher than any other reported 2D material with a sheet carrier concentration over 10^13^ cm^−2^.

Focused plasma treatment of O_2_, N_2_, and H_2_ have been employed on solution-processed In_2_O_3_ TFTs and improved performance and stability in air and under PBS/NBS. Focused O_2_ leads to improvement from 1.35 to 2.48 cm^2^/Vs. Focused N_2_ leads to TFT mobilities of up to 5.06 cm^2^/Vs, and an N_2_-H_2_-O_2_ mobility of 3.8 cm^2^/Vs [180,181]. Note that the TFT maximum process temperature was 250 °C. X. Li et al. reported the use of NH_3_ treatment on solution-processed In_2_O_3_ TFTs and showed that the treatment resulted in H doping, less contamination, and more V_o_, leading to mobilities of 3.62 cm^2^/Vs at 300 °C [182].

N_2_O plasma treatment can represent an effective alternative to O_2_ plasma treatment. Rabbi et al. explained that N_2_O plasma treatment decreases Vo and -OH groups, leading to a decrease in hysteresis but also to the increased density of the material, which O_2_ was not reported to do [183]. The benefit of N_2_O plasma is counterbalanced by the power to be correctly monitored [184].

Finally, in terms of plasma treatment, fluoride-based plasma treatments have shown drastic improvement over other treatments, and the effect of F in In_2_O_3_ has been supported by DFT calculations. CF_4_ helps decrease the Fermi level because the F can occupy an oxygen vacancy site and decrease the carrier concentration, according to the following equation [185].

V_o_^2+^ +2e^−^ + F. -> F_0_^+^ + e^−^

So, F can act on the carrier concentration, monitoring the Vth. The authors used the effects to manufacture inverters, one TFT being in the depletion mode (without CF_4_ treatment), and the other one in the enhancement mode (with CF_4_ treatment). The gain of the inverter was higher than 60.

**Figure 9:**
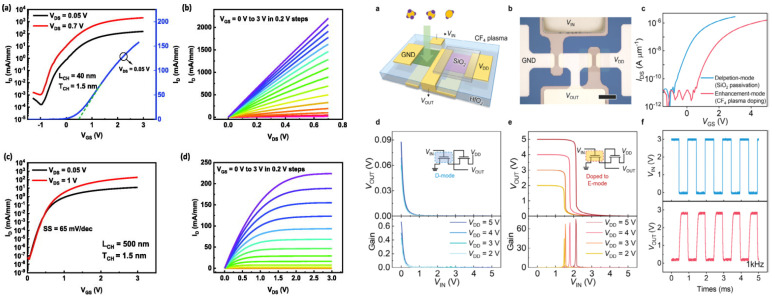
Effect of plasma treatment on In_2_O_3_ TFT. **Left**: effect of oxygen plasma on TFT IV curves. Reprinted from [177] Charnas, A.; Si, M.; Lin;Z.; Ye, P. Enhancement-mode atomic-layer thin In_2_O_3_ transistors with maximum current exceeding 2 A/mm at drain voltage of 0.7 V enabled by oxygen *Appl. Phys. Lett.* **2021**, *118*, 052107, with the permission of AIP Publishing. **Right**: Effect of CF_4_ plasma treatment and application to circuits; ©2024 Wiley-VCH GmbH, Permission to reproduce from [185] granted by John Wiley and Sons.

J. Li et al. showed that stable F_o_F_i_ defects can be formed and passivate the oxygen vacancy, therefore decreasing the carrier concentration, and leading to more stable TFTs [186]. The high bond energy of In–F (506 kJ/mol) being higher than In–O bonds (346 kJ/mol) can also participate in the enhancement of stability of the In_2_O_3_ TFTs [186] with a variation of only 0.05 V under PBS. J. Zhang et al. demonstrated that a 1 min CF_4_/N_2_O plasma treatment can modify the TFT mode (change from depletion to enhancement), while O_2_ does not significantly modify the Vth [187]. Let us note that F should behave as an amorphization agent in amorphous In_2_O_3_ but also in crystalline In_2_O_3_ [188]. An example of the use of CF_4_ plasma treatment is shown in Figure 9.

### 6.3. Source and Drain

Correctly assessing the mobility is important and has been reported many a times for a-Si:H and IGZO TFTs, and correctly designing the semiconductor and source/drain (S/D) overlap is one way to correctly assess the mobility. With correct assertion, T. Takahashi et al. fabricated In_2_O_3_ TFTs with a GaO_x_ passivation layer and obtained a mobility of 100 cm^2^/Vs [189]. Let us note that the contact resistance is primordial to the TFT performances. For example, JH Lee et al. showed that at high V_GS_, the contact resistance can represent almost 98% of the resistance of the TFT [190].

J Y Lin et al. [191] demonstrated the dependency of the metal S/D on the channel length. They explained that during deposition, oxygen vacancies may form locally near/at the interface with the contacts, because the metal contact could create a metal–oxygen bond more efficiently than In-O.

On the other hand, YY Pan and coworkers [192] showed the importance of controlling the temperature during the process. The heat during the deposition may affect the resistivity of the underlying In_2_O_3_ and would especially affect scaled devices. Also to consider is the latent heat of condensation of the metal: the lower the latent heat, the lower the effect of L_ch_ on Vth. The two would affect oxygen vacancies in In_2_O_3_.

**Figure 10:**
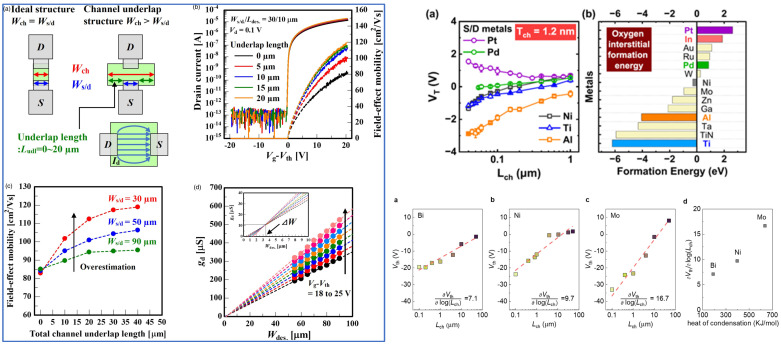
Source/Drain contact design. **Left**: channel and S/D design, figure under CC BY license, reproduced from [189]. **Top right**: effect metal on V_th_ and oxygen interstitial formation energy, 1557-9646 © 2025 IEEE. All rights reserved, including rights for text and data mining, and training of artificial intelligence and similar technologies. Personal use is permitted, but republication/redistribution requires IEEE permission. See https://www.ieee.org/publications/rights/index.html (Accessed on 8 September 2025) for more information, reprinted with permission from [191], Lin, J.; Niu, C.; Lin, Z.; Lee, S.; Kim, T.; Lee, J.; Liu, C.; Lu, J.; Wang, H.; Alam, M.; Jeong, C.; Ye, P. Analyzing the Contact-Doping Effect in In_2_O_3_ FETs: Unveiling the Mechanisms Behind the Threshold-Voltage Roll-Off in Oxide Semiconductor Transistors IEEE Trans. Elec. Dev., **2025**, 72, 3004–3011. **Bottom right:** Vth as a function of channel length depending on the metal, figure under CC BY 4.0 license, reproduced from [192].

Finally, let us note another way to improve the performances of a TFT by its S/D, which is by the introduction of a tungsten polyoxometalate (POM) layer between the S/D and the In_2_O_3_ layer. The POM layer facilitated the electron injection from the S/D to the In_2_O_3_ layer, the mobility could have increased from 3.9 to 10.8 cm^2^/Vs and decreased the off current by ~2 orders [193]. Figure 10 below gathers a few strategies explained in this section.

### 6.4. Homojunctions

Double layer channels or homojunctions have been introduced as channel regions for TFTs because they offer the ability to control carrier concentration and channel protection from the environment. As for In_2_O_3_, nitrogen incorporation during the deposition can positively alter the TFT properties. YC Cheng et al. [194,195] fabricated an IGO layer without N_2_ above an IGO layer manufactured with N_2_ flow, leading to an enhancement of mobility to 25 cm^2^/Vs compared to a single layer of IGO having a mobility of 14 cm^2^/Vs. A 280 °C solution-processed In_2_O_3_:F/In_2_O_3_ offers an improvement in TFT performances from a single-layer In_2_O_3_ by increasing the mobility from 0.3 to 5.69 cm^2^/Vs when the F doping was 15%. The Vth was also slightly shifted from 7.92 down to 5.80 V, and an improvement in the SS from 1.01 to 0.27 V/dec. With Al_2_O_3_ as the GI, the TFT performance increased further up to 31.4 cm^2^/Vs [196]. Oxygen-poor In_2_O_3_ and oxygen-rich In_2_O_3_ used as homojunctions by M. Zheng et al. reached a TFT mobility of 32.5 cm^2^/Vs by controlling the oxygen flow during deposition [197].

### 6.5. Dual Gate Structure

Dual gate structure TFTs are well known to have improved parameters compared to their single gate TFT counterparts. Among others, when using both gates, the structure offers an improved current, leading to an increased mobility, but also offers a higher stability against various stresses.

A series of 7 nm-thick amorphous 1% W-doped In_2_O_3_ layers used in dual gate TFTs can decrease the DIBL in a small channel TFT (100 nm) and increase the current and therefore the mobility (~20 cm^2^/Vs). Also, the stability was drastically improved under NBS (from −0.8 V down to less than −0.2 V) [198]. The dual gate structure can also be used to decrease the negative Vth shift under PBTS observed in top gate TFTs [199]. J. Kwak et al. demonstrated the use of machine learning to improve TFT characteristics against PBTI. The authors showed by careful design of a dual gate structure that IWO TFTs can show high stability against PBTI [200]. By obtaining higher currents, J. Sun et al. demonstrated dual gate a-IGO TFTs having mobilities of 52 cm^2^/Vs, but more importantly they showed similar current to poly-Si TFTs, that could lead to higher performance LTPO devices [201]. C. Ye et al. used O_3_ plasma treatment on ALD-manufactured dual gate IGO TFTs and obtained a g_m_ of 1008 µS/µm and an SS of 63 mV/dec. O_3_ helped decrease the defects without decreasing the carrier concentration [202]. The dual gate structure is also a way to reduce remote Coulomb scattering (RCS) as observed by the almost temperature independent field effect mobility of the dual gate structure IGO TFT proposed by C H Choi et al. [203].

### 6.6. Interaction with Organic Materials

Other strategies to improve TFT initial characteristics and stabilities could be to consider the use of organic materials. TIPS pentacene was deposited on top of solution-processed In_2_O_3_ and enhanced the electron percolation conduction but also acted as a passivation layer. When the S/Ds were deposited on top of In_2_O_3_, they obtained enhancement mode TFTs, and when deposited on top of TIPS pentacene, they obtained depletion mode TFTs, both having mobilities of 6.3–6.4 cm^2^/Vs [204]. ST Wang et al. showed the reduction ability of viologens, inducing high carrier concentration in 2D In_2_O_3_ TFTs, and therefore a negative Vth shift [205]. Organic passivation, like PI passivation [206] can help retain the TFT characteristics over time as ZL Zhang et al. showed.

### 6.7. Passivation

While we introduced various results of TFT performances with or without passivation, let us discuss here some aspects that could be considered for effective passivation of In_2_O_3_ TFTs. As mentioned before ALD-manufactured In_2_O_3_ TFTs suffer from negative shifts under PBS and positive shifts under NBS, so strategies to reduce this effect have been investigated. KKH Lin et al. used H_2_O_2_ treatment and HfO_2_ as a passivation on 2 nm-thick In_2_O_3_ TFT and obtained high thermal and negligible Vth shifts under thermal stress of 250 °C for a channel length as low as 50 nm [207]. A similar approach was used by T. Gao et al. They showed similar results when their devices were used at a temperature up to 85 °C [208]. PR Ghediya et al. studied the effect of various layers as passivation layers and demonstrated that the most effective passivation layer is the one which has the lattice constant the closest to In_2_O_3_, namely Y_2_O_3_ and Er_2_O_3_ [209]. With these passivation layers, the TFT demonstrated a slight decrease in mobility (from 100 down to 70–80 cm^2^/Vs) but demonstrated the best stability under NBS and PBS. Multiple stack layers of In_2_O_3_/HfO_2_ have been investigated and demonstrated the viability of 1 nm-thick deposited layers [210].

## 7. Applications

We will first introduce 1D and 0D based In_2_O_3_ TFTs, sensors, then discuss about various applications, from neuromorphic applications, heterostructures leading to 2DEG, and 3D integrations. In this part, we do not discuss applications like inverters or common electronic circuits.

### 7.1. One-Dimensional and Zero-Dimensional Use of In_2_O_3_ in TFTs

**Figure 11:**
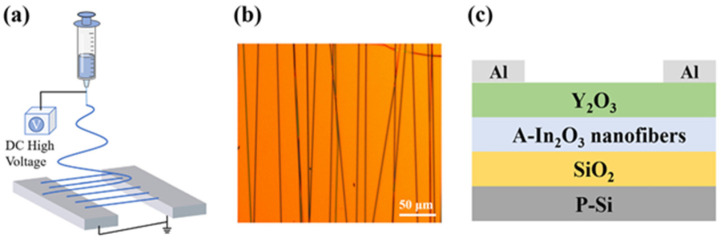
In_2_O_3_ nanofiber fabrication process and TFT. Reprinted from [213], with the permission of AIP Publishing.

There are not many reports on the use of In_2_O_3_ quantum dots (QD) in TFTs. Let us note the reports of S. L. Swidher et al. which demonstrated an In_2_O_3_ QD TFT with a mobility reaching 10 cm^2^/Vs and an on/off ratio of 10^6^ [211]. In_2_O_3_ nanofibers are usually made by the low temperature budget electrospinning process. In_2_O_3_ nanofibers have been fabricated and implemented into TFTs. An example of In_2_O_3_ the nanofiber TFT fabrication process is shown in Figure 11. Y. Ding et al. fabricated flexible In_2_O_3_ nanofiber TFTs and obtained a mobility of 14.64 cm^2^/Vs and an SS value of 75 mV/dec [212].

L. Tian et al. reported fully passivated In_2_O_3_ nanofiber TFTs with a mobility reaching 18.2 cm^2^/Vs [213]. Other works on the coverage of nanofibers showed that a 10% fiber density could lead to mobilities of 5.5 cm^2^/Vs [214]. At a low thermal budget of 35 °C, D. Zhang et al. could fabricate flexible devices on PI with a mobility of 7.82 cm^2^/Vs [215]. Let us note the fabrication of As-doped In_2_O_3_ NW for TFTs and display applications having mobilities reaching 1500 cm^2^/Vs [216].

Finally, let us mention the fabrication of 3% Pr-doped In_2_O_3_ nanofiber TFTs by Z. Peng et al. The TFTs demonstrated mobility of 6.92 cm^2^/Vs, and high stability under NBIS [217].

### 7.2. Sensors

In_2_O_3_ devices have been used also as sensors. NO_2_, H_2_S, and CO_2_ [218] are the main gas reported to be detected. We invite the reader to refer to [219] for a more detailed review on the matter. We herein include only a few recent strategies and advances. Solution-processed TFTs with 1% PEI doping demonstrated superior sensing properties than In_2_O_3_. A detection limit as low as 10 ppb could be achieved thanks to the higher carrier concentration with PEI doping [220].

Integration of MXene or graphene or heterostructures of IGZO/In_2_O_3_ also enhances the NO_2_ sensing abilities [221,222,223] and can lead to a detection limit as low as 1 ppb. Recently, G. Jung et al. showed the possibility of effectively detecting H_2_S or NO_2_ by controlling the surface oxygen concentration and modifying the chemisorption of the surface by simply applying voltage [224]. N_2_O/H_2_S gases can also be effctively detected by using a floating gate device [225].

In_2_O_3_ TFTs have also been used as ion detecting devices, notably for detecting pH [226,227,228,229], iodine [230], nitrite, [231] or as biosensors [232,233,234] to detect low concentrations of RNA, DNA, or ions like K+ and Na+.

### 7.3. Neuromorphic Applications

Let us first describe a few properties to understand the concept of a synapse TFT. A presynaptic pulse acts on the TFT which will respond by an excitatory post-synaptic current (EPSC), from which we can establish the difference from short-term plasticity (STP) to long-term plasticity (LTP). For a counterclockwise (clockwise) hysteresis, the ESPC is caused by a positive (negative) spike. Finally, paired-pulse facilitation (PPF) and paired-pulse depression (PPD) properties can be established and they define the quality of the synaptic retention.

In_2_O_3_ and doped In_2_O_3_ TFTs have been used in various neuromorphic applications. The concept lies on the memory effect induced by a ferroelectric material, either by the gate insulator or the semiconductor. The clockwise or anticlockwise hysteresis would be used as a memory effect.

Yet, In_2_O_3_ has no ferroelectric behavior by itself. As explained by R. Dobhal et al. [235] a memory window can nonethelesss be induced by a dipole effect in a SiO_2_ gate insulator. The effect can be tuned by the applied electric field, so a thinner SiO_2_ layer would allow a larger anticlockwise hysteresis. To create a hysteresis window in In_2_O_3_-based TFTs, a ferroelectric dielectric could be used. For example, HfZrO dielectric was used to manufacture ferroelectric (FE) ITO TFTs, further used in artificial neural networks using the MNIST database for training [236,237,238]. Y. Du et al. demonstrated that the introduction of Li in a Gd oxide gate insulator can turn a In_2_O_3_ TFT from being hysteresis-free to having a hysteresis window and could be used for synaptic applications [239].

On the other hand, H Song et al. used the oxygen vacancy modulation in solution-processed multiple-layered IGO TFTs. With a channel made of various layers with different Ga content, they correctly observed LTP and LTD. They used their TFT for visual recognition [240].

**Figure 12:**
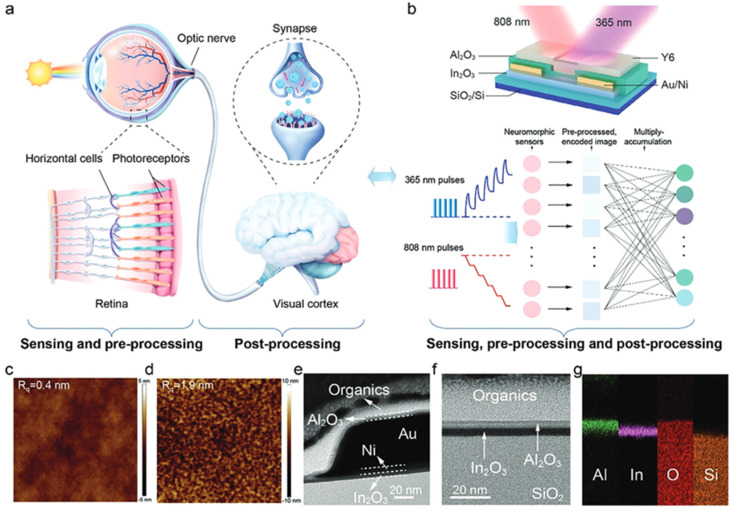
Example of a neuromorphic sensor based on light using In_2_O_3_ TFT © 2023 Wiley-VCH GmbH, Permission to reproduce from [241] granted by John Wiley and Sons.

D. Li. et al. used no ferroelectric effect [241]. They fabricated In_2_O_3_ TFTs with Al_2_O_3_/Y6 on top. Y6 is used as a NIR absorber and the authors used their layer engineering abilities to induce a negative photocurrent under NIR and positive photocurrent under UV, by either using or electron trapping in Al_2_O_3_ or generating electrons from oxygen vacancies in In_2_O_3_, respectively. The concept is shown below in Figure 12.

H. Lee et al. demonstrated the use of a ferroelectric layer not as the gate insulator but as the substrate on which the channel layer would be deposited [242].

Let us note that O. Phadke et al. demonstrated by analyzing low noise frequency characteristics that ferroelectric TFTs were more prone to degradation under DC stress than their non-ferroelectric counterparts [243].

Finally, let us note that several reports showed the use of electrolyte-gated synaptic transistors. Lactose-citric acid, ion gel GI, AlLiO, and LiClO4/PEO layers are examples used in synaptic TFTs [244,245,246,247,248]. The direction of the hysteresis can be modified and therefore the behaviors can be easily monitored.

### 7.4. 2DEG

Another way to alleviate TFT degradations or to increase their mobility is through the concept of double layers, forming a heterojunction in the channel region. Various heterojunctions have been introduced, and the main concept lies on the formation of a 2DEG layer at the interface of the heterojunction because of the discontinuity of the CB, which can therefore lead to higher mobility. H. Faber et al. reported the concept in 2017, by using a solution-processed 13 nm-thick In_2_O_3_/ZnO heterojunction. The heterostructure had a mobility of 45 cm^2^/Vs, whereas ZnO had a mobility of ~1 cm^2^/Vs or less. The heterojunction TFT also demonstrated no temperature dependency, which indicates band-like electron conduction [249].

**Figure 13:**
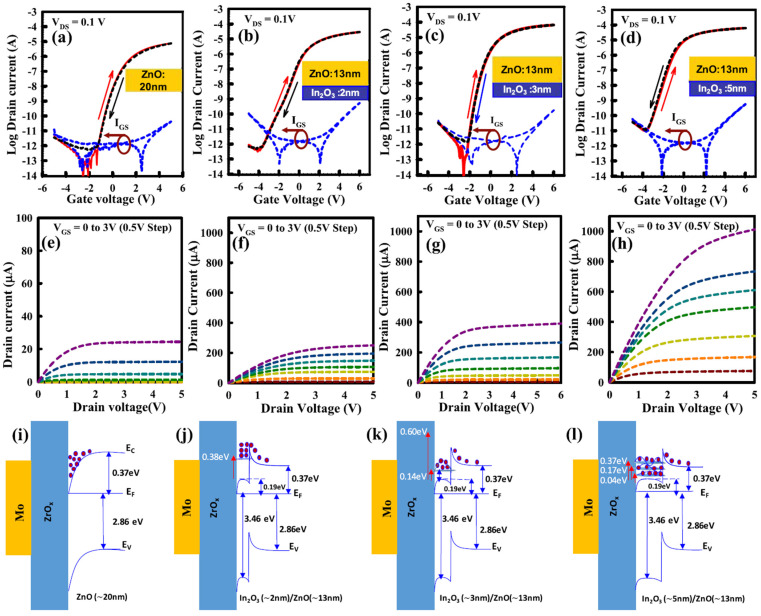
2DEG structure obtained in In_2_O_3_/ZnO stack based TFTs. (Reprinted with permission from reference [252]. Copyright 2024 American Chemical Society).

Z. Li et al. demonstrated that at a fixed thickness, the Vth (mobility) decreases (increases) with indium oxide thickness, while the SS is rather constant when In_2_O_3_ is introduced. Their optimal condition reached a mobility of 44.7 cm^2^/Vs for an I_ON_/I_OFF_ of 10^9^ [250]. On the other hand, W.S. Al Ghamdi et al. showed that for a minimum thickness of 5 nm, and whatever the thickness of ZnO is, no significant change in the mobility could be observed in their TFTs, but this had an effect on the Vth [251]. Saha et al. demonstrated the importance of controlling the thickness of In_2_O_3_ [252]. An optimum thickness corresponds to the optimum quantum well confinement and control of energy levels in it. With an optimum thickness of 3 nm for In_2_O_3_, they obtained a µ_sat_ of 97 cm^2^/Vs, as shown below in Figure 13.

Using ALD supercycles, Z. Wang et al. fabricated In_2_O_3_/ZnO multistacks and demonstrated polycrystalline IZO layers. An IZO TFT reached the mobility of 177.1 cm^2^/Vs with L_ch_ = 2.5 µm [253]. Li doping into ZnO could also modulate the carrier concentration in spin-coated oxide bilayers In_2_O_3_/ZnO as reported by D. Khim et al. They could obtain a mobility of 10.7 cm^2^/Vs [254]. Blade coating of In_2_O_3_/ZnO/In_2_O_3_ on AlO_x_ GI for flexible applications reached a mobility of 10.7 cm^2^/Vs, whereas on SiO_2_ the mobility could reach 38.2 cm^2^/Vs [255].

Other than the In_2_O_3_/ZnO heterojunction channel layer, other heterojunctions have been investigated demonstrating various advantages over a single layer. For example, ZnO/IGO demonstrated similar improvement in TFT performances, with a mobility reaching 63.2 cm^2^/Vs with the formation of a 2DEG layer at the interface [256]. T Huang and coworkers used ITO/IGZO and demonstrated a drastic decrease in the Vth, a shift of ~80% under PBS, due to the barrier at the interface leading to a decrease in electron trapping from the top surface [257]. CY Park et al. also revealed a thickness dependency of the ITO layer obtaining a similar mobility of ~31.9 cm^2^/Vs for a 4 nm thick ITO layer with IGZO stacked layer [258]. They obtained a mobility of ~58.2 cm^2^/Vs when Al_2_O_3_ was used as the GI.

Polycrystalline IGO/IGZO also was investigated, leading to mobilities of 95.7 cm^2^/Vs compared to IGO alone (61.4 cm^2^/Vs). The double layer TFT also showed a high on/off ratio of 10^10^ [259]. Using the same strategy, IGO/AIGO resulted in mobilities of 43 cm^2^/Vs [260]. Solution processed ITO/IGO heterojunction TFT reached a mobility of 17.44 cm^2^/Vs while having Vth shift under PBS and NBS of 0.1 and −0.15 V, respectively [261].

R Chen et al. fabricated IGO homojunction channel layer TFT [262]. Let us remark that the field effect mobility graph can provide information related to the conduction mechanism via the equation.

$$\mu_{FE}=K{\left(V_{GS}-V_{TH,P}\right)}^{\gamma }$$

where γ is an exponent related to the conduction mechanism and V_P_ defines the onset voltage for the percolation mechanism to operate. This equation has been used by other researchers, with more accurate parameters (e.g., temperature dependency) [263,264,265]. When γ is 0.4 or above, the main mechanism is the trap limited conduction, and when γ is ~0.1, the main mechanism is percolation conduction [262]. Their optimized device achieved a μ_FE_ = 77.5 cm^2^ V/Vs with ΔV_th_ = −0.61 V (NBS), +0.07 V (PBS). By DFT and low frequency noise analysis, J. He et al. demonstrated that the bilayer can reduce the trap density, explaining the higher mobility but also the smaller hysteresis. With an HfO_2_ GI they obtained a mobility of 67.5 cm^2^/Vs [266]. The introduction of Sn into IGO in an In_2_O_3_/IGO channel, led to a smaller formation of oxygen vacancies, leading to higher carrier concentration and could explain, in part, a high mobility of 43.6 cm^2^/Vs [267]. Q. Zhang et al. obtained a mobility of 47.7 cm^2^/Vs with a solution-processed In_2_O_3_/IGZO double layer and an annealing by VIS-IR irradiation [268]. Let us note that combustion process was also investigated for double layer channels, and while seperated In_2_O_3_ and IGO lead to mobilities of 3.2 and 0.5 cm^2^/Vs, the double layer had a mobility of 2.6 cm^2^/Vs but a higher on/off ratio and a Vth shift close to 0 V [269].

**Figure 14:**
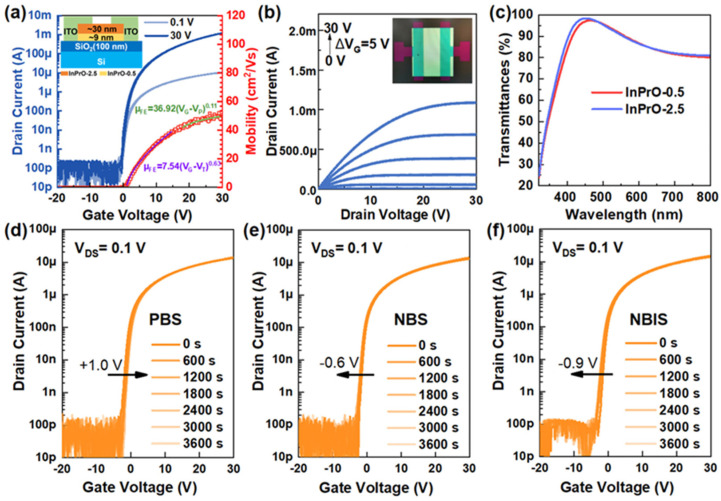
Pr-doped In_2_O_3_ homojunction TFTs and their characteristics. Reprinted from [265]: Wu; J.; Guo; M.; Wu, Q.; Han, S.; Lu, X.; Liang, X.; Liu, C. Achieving high mobility and enhanced illumination stability in InPrO homojunction thin-film transistors *Appl. Phys. Lett*. **2025**, *126*, 093502 https://doi.org/10.1063/5.0246425, with the permission of AIP Publishing.

The Ni-doped In_2_O_3_/Ga_2_O_3_ bilayer TFTs reached a mobility of 47 cm^2^/Vs and were used as photodetectors [270]. With Sm, W. Xu et al. fabricated a double layer of In_2_O_3_/InSmO TFTs. The layer had c-axis aligned crystallinity. The TFT showed a high mobility of 40.3 cm^2^/Vs. Let us note that the best stability was obtained for the 30% doped Sm layer [271]. Finally, Pr-doped In_2_O_3_ homojunctions have also been reported. The authors obtained a mobility of ~50 cm^2^/Vs, Vth shift of 1 V, −0.6, and −0.9 V for PBS, NBS, and NBIS during 1 h, respectively [265]. Figure 14 below show Pr-doped In_2_O_3_ double layer characteristics.

Also, crystallized IGO/IGZO double-layered TFTs could reach a mobility of 95.7 cm^2^/Vs, with improved NBS stability compared to the single-layered IGO TFTs [259].

### 7.5. Three-Dimensional Integration

Scaling down the In_2_O_3_ has opened the path to various applications. One of them is DRAM. Process-wise, devices need to withstand process temperatures of ~600 °C. HJ Oh et al. [272] reported IGO devices withstanding process temperatures of up to 700 °C, and showing mobilities of ~68.7 cm^2^/Vs. SH Ryu et al. also reported c-axis oriented IGO devices with a mobility of 128.2 cm^2^/Vs and withstanding 700 °C [273]. On the other hand, JY Liu et al. fabricated 2 nm-thick In_2_O_3_ TFTs with Lch of 100 nm that withstand a process temperature of 600 °C [274]. On the other hand, the 2 transistor gain cell (2TGC), a structure suitable with BEOL applications, has been demonstrated with IWO devices [275].

Integration, not only by scaling but by stacking, has also been demonstrated. S: Yuvaraja et al. demonstrsated the possibility to fabricate 10 stacks of In_2_O_3_-based TFT with a mobility of ~15 cm^2^/Vs and demonstrated the operation of unipolar inverters [276].

**Figure 15:**
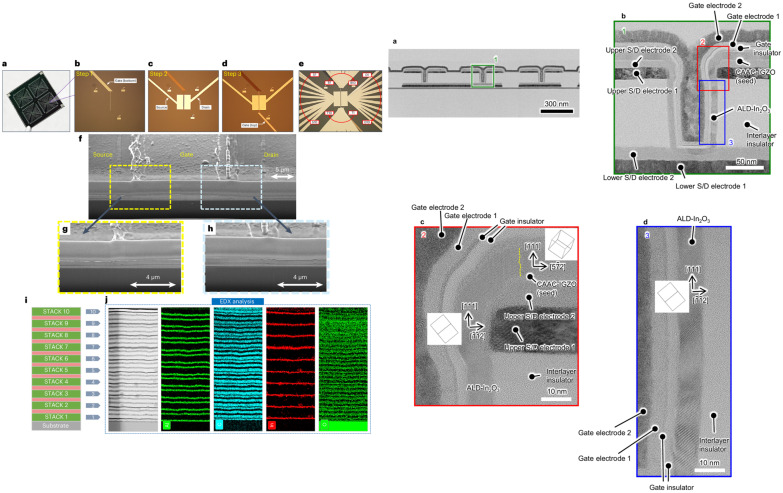
Three-dimensional integration of In_2_O_3_ transistors. **Left**: 10 stack TFTs, figure under CC BY 4.0, reproduced from [276], **right**: integration of In_2_O_3_ devices, figure under CC BY license, reproduced from [277].

S. Yamazaki et al. fabricated vertical transistors, using single crystalline In_2_O_3_ as the channel with a low off current of 10^−21^ A μm^−1^ and an SS of 86.7 mV/dec [277]. Examples of 3D integration are shown in Figure 15. Z Zhang et al. demonstrated In_2_O_3_ vertical based ferroelectric devices (using HZO) with an endurance of 10^9^ cycles and more than 10 years of retention [278]. Scaling down and integrating devices as BEOL, the FEOL devices may be negatively affected by any self-heating effect from the BEOL devices. So, solutions integrating separation layers are also of interest [279]. As a possible integration, In_2_O_3_ TFTs were associated with GaN HEMT, demonstrating a temperature stability of up to 125 °C and showing a high drain current of 50 mA/mm. The TFT Vth was controlled by the gate material: iridium for enhancement, and aluminum for depletion mode [280]. Finally, let us note gate all around (GAA) devices made of W-doped In_2_O_3_, reached mobilities of ~22 cm^2^/Vs [281].

## 8. Conclusions

We have given a deep outlook on In_2_O_3_ and its use in TFTs, scaled devices, and future electronics applications. Undoped or doped In_2_O_3_ TFTs may represent the future of devices in large area electronics. In_2_O_3_ has a direct optically forbidden bandgap of 2.9 eV, and an optically allowed transition of 3.7 eV. The material can reach carrier concentrations in the 10^20^–10^21^ cm^−3^ range through the control of oxygen vacancies and by substitutional doping. In place of Sn doping, transition metals and hydrogen have the ability to enhance the carrier concentration and mobility without a significant trade-off with the optical properties of the material. Applied to TFTs, H doping can lead to high mobilities of 100 cm^2^/Vs. Other doping schemes (Hf, Mo, and others) control the carrier concentration and may drop the mobility when doping is too high. ITO and IGO seem to be the most promising materials with mobilities above 30 cm^2^/Vs, and reaching 80 cm^2^/Vs. Other strategies like plasma treatment, heterostructures, and dual gate TFTs can also enhance the TFT mobility. Nonetheless, let us note the lack of understanding and theoretical background of the plasma effect on the surface layer of In_2_O_3_, e.g., why N_2_O plasma treatment would be better than O_2_. The material on which In_2_O_3_ is deposited may have an impact on the TFT characteristics (in terms of hysteresis and mobility for example) and should be carefully chosen. Careful attention should be paid during processes, especially when choosing and depositing the S/D metal electrode, as excess oxygen vacancies could be created near/at the interface. In terms of passivation, while high k dielectrics have been used successfully, Y_2_O_3_, or ErO_2_ should provide the best TFT passivation because of good lattice match with In_2_O_3_. Enhancement of the TFT NBIS stability can be achieved through doping with lanthanide, and more specifically with Tb or Yb. From bulk 3D down to 0D, all dimensions have been tried, but 2D of 2 nm or less thin layers of In_2_O_3_ or doped In_2_O_3_ seem to lead to the best TFT performances. There have been various demonstrations of BEOL applications, neuromorphic applications, DRAM, vertical integrations, and various sensors. Let us note that while the vacuum process has led to the most investigations, non-vacuum solutions like spin-coating and spray-coating, or the more recent metal liquid printing are competitive in terms of thermal budget and in terms of the achievable TFT mobility. In terms of the solution process, the nitride precursors have been widely investigated, while few others based on chloride, for example (as for sprayed Ga-doped In_2_O_3_), seem to provide suitable results for large area electronics. ALD has seen the development of many precursor materials, whereas in the solution process, in-house developed precursors or precursors other than nitride and chloride-based precursors are still lacking. Also, investigations to develop p-type In_2_O_3_ are still to be led in order to fabricate next generation CMOS and electronics.

## Figures and Tables

**Table 1:** Comparison of typical parameters of indium oxide-based TFTs with various processes.

Preparation Method	Process Temperature (°C)	Mobility (cm^2^/Vs)	L_ch_	Best Dopant
Spin-coating	200–300	0.1–10	>µm	Sn, Ga
Spray-coating	>350	30–50	Down to ~1 µm	Ga
Vacuum process (ALD)	<250	80–150	Down to nm	W, H
SPC	<250	~100	>µm	H

**Table 2:** Summary of the dopants investigated and their use in TFT applications.

Dopant	Purpose
H	Mobility, Crystal size
Lanthanides	NBIS stability
Ga	Mobility, amorphization
Sn	Mobility, high frequency operation
W, Li	Mobility
Mo, Hf, Sb, Si, B, S	Amorphization V_th_, On/Off ratio, S.S. control and improvement Reduction of carrier concentration
PEI, PEIE	Mobility

## Data Availability

No new data were created or analyzed in this study.
